# Synthesis, Structure, and Antiproliferative Action of 2-Pyridyl Urea-Based Cu(II) Complexes

**DOI:** 10.3390/biomedicines10020461

**Published:** 2022-02-16

**Authors:** Kirill K. Geyl, Sergey V. Baykov, Stanislav A. Kalinin, Alexandr S. Bunev, Marina A. Troshina, Tatiana V. Sharonova, Mikhail Yu. Skripkin, Svetlana O. Kasatkina, Sofia I. Presnukhina, Anton A. Shetnev, Mikhail Yu. Krasavin, Vadim P. Boyarskiy

**Affiliations:** 1 Institute of Chemistry, Saint Petersburg State University, 199034 Saint Petersburg, Russia; kirillgeyl@outlook.com (K.K.G.); tat.sharonowa2016@yandex.ru (T.V.S.); skripkin1965@yandex.ru (M.Y.S.); s.kasatkina@spbu.ru (S.O.K.); sonya.presnuxina.98@mail.ru (S.I.P.); m.krasavin@spbu.ru (M.Y.K.); v.boiarskii@spbu.ru (V.P.B.); 2 Medicinal Chemistry Center, Togliatti State University, 445020 Togliatti, Russia; brglab@tltsu.ru (A.S.B.); mcc@tltsu.ru (M.A.T.); 3 Pharmaceutical Technology Transfer Center, Yaroslavl State Pedagogical University Named after K.D. Ushinsky, 150000 Yaroslavl, Russia; a.shetnev@yspu.org

**Keywords:** metal complexes, cytotoxicity, ureas, anti-cancer drugs, lung cancer

## Abstract

Relying on a recently suggested protocol that furnishes convenient access to variously substituted 2-pyridyl ureas, twelve hitherto unknown Cu(II) complexes have been synthesized in the present work and their structures were evaluated by elemental analysis, HRMS, IR spectroscopy, and X-ray diffraction study. Two structural motifs ([Cu(L)_2_Cl]^+^[Cl]^−^ or (Cu(**L**)_2_Cl_2_) depending on the substitution pattern on the 2-pyridine fragment were revealed. In addition, antiproliferative action of the obtained compounds have been investigated against lung cancer cell lines (A549, NCI-H460, NCI-H1975), and healthy WI-26 VA4 cells were used to monitor non-specific cytotoxicity. Two nitro-group substituted complexes Cu(**U3**)_2_Cl_2_ (IC_50_ = 39.6 ± 4.5 μM) and Cu(**U11**)_2_Cl_2_ (IC_50_ = 33.4 ± 3.8 μM) demonstrate enhanced activity against the drug resistant NCI-H1975 cells with moderate selectivity toward normal WI-26 VA4 cells. The antiproliferative mechanism of cell death underlying the growth inhibitory effect of the synthesized complexes was studied via additional experiments, including the cell cycle analysis and the apoptosis induction test. Reassuringly, certain 2-pyridyl urea-based Cu(II) complexes exerted cell line-specific antiproliferative effect which renders them valuable starting points for further unveiling the anticancer potential of this class of coordination compounds.

## 1. Introduction

Being one of the leading causes of mortality worldwide, cancer diseases represent a tremendous challenge to the healthcare system around the globe [1]. In fact, the genesis and progression of different malignancies are extremely complex and the development of multidrug-resistant phenotypes often leads to recurrence in the neoplasms initially responsive to the therapy [2]. This is true for either conventional cytostatic agents, including metal-based drugs, or targeted cancer treatments that deal with signaling and metabolic vulnerabilities in malignant cells [3]. In this context, the design of novel anticancer therapeutics is the important goal for chemical science.

Metal-based anticancer agents are among the most successful therapeutic agents, as evidenced by their frequent prescription to patients since their discovery [4]. In fact, there is a plethora of reports in recent years on the transition metal complexes proving highly efficient as anticancer agents [5,6,7,8,9,10,11]. Among other metal-based anticancer compounds, Cu(II) complexes recommended themselves as promising cytostatic agents due to Cu(II) plays a crucial role in cell growth and metabolism [12,13,14,15,16,17,18,19]. Although there is a variety of mechanisms underlying the Cu-based drugs’ anticancer effect, generally, it is based on the interplay between the metal and the organic ligand. For example, these agents act as chelators, displace other ions from the enzyme binding sites, trigger intracellular copper accumulation, cytotoxicity, and activate apoptosis inhibitor factor (XIAP). Some of them are known to inhibit proteasome and interfere with DNA through binding along with the major or the minor DNA grooves and intercalation. Moreover, copper-based drugs are known to inhibit topoisomerases as well as generate reactive oxygen species (ROS) producing oxidative damages in the cytoplasm, mitochondria, and DNA [12]. Therefore, the optimization of ligand structures is one of the main approaches to developing new Cu(II) complexes with anticancer effects.

It is widely known that 2-pyridyl urea derivatives are *N,O*-chelate ligands capable of efficiently forming complexes with transition metals [15,20,21,22,23,24,25,26], among which Cu(II) holds a unique position. We have noticed that only a handful of Cu(II) incorporating 2-pyridyl urea complexes have been reported to date. Moreover, while structural data and spectroscopic properties of these uncommon complexes have been investigated, none of the reports contained biological activity data. This disappointing void is primarily related to the low synthetic availability of 2-pyridyl ureas and the need to utilize toxic reagents in their preparation process [27,28,29,30,31]. To address this latter problem, we recently came up with a simple protocol for the preparation of diverse *N*,*N*-dialkyl-*N*′-(pyridin-2-yl)-ureas via acid-catalyzed reaction of pyridine-*N*-oxides with dialkylcyanamides [32,33,34,35].

The structure and reactivity (including coordination with Pt(II)) of these compounds were studied [36,37,38]. In the present study we employed *N*,*N*-dialkyl-*N*′-(pyridin-2-yl)-ureas, thus obtained, to synthesize novel Cu(II) complexes in the context of the global drug discovery efforts for metal-based tumor-suppressing agents. Herein, we report the preparation of 12 hitherto undescribed Cu(II) complexes incorporating 2-pyridyl urea backbone, their structural studies as well as the evaluation of their in vitro anticancer activities.

## 2. Materials and Methods

### 2.1. General

The preparation and characterization of starting pyridine-*N*-oxides as well as **U1–12** are described previously [33,34,37]. CuCl_2_, other reactants, and solvents were obtained from commercial sources. High-resolution mass-spectra (HRMS) were measured on Bruker Maxis HRMS-ESI-qTOF (Electrospray Ionization, ESI; Bruker, Billerica, MA, USA); a MeOH was used as the solvent. The instrument was operated at positive ion mode using *m/z* range of 50–3000. The capillary voltage of the ion source was set at –4500 V (ESI^+^) and the capillary exit at +(70–150) V. The nebulizer gas pressure was 0.4 bar and drying gas flow was 4.0 L/min. The most intensive peak in the isotopic pattern is reported. Infrared spectra (4000–400 cm^–1^) were recorded on a Shimadzu FTIR 8400S (Shimadzu, Kyoto, Japan) instrument in KBr pellets. The C, H, and N elemental analyses were carried out on a Leco TruSpec Micro CHNS analyzer (LECO, St. Joseph, MI, USA). TGA was performed on a ca. 2 mg samples of Cu(II) complexes by using a Netzsch TG 209 F1 Libra thermal analyzer (Netzsch, Selb, Germany). The samples were dried under a vacuum at 50 °C before heating from 40 to 450 °C at a heating rate of 10 K min^–1^. A flow rate of 10 mL min^–1^ of dry argon was used to purge the samples. Molar conductivity was measured with an Elico digital conductivity bridge model CM-88, using a freshly prepared DMSO solution of the complexes.

### 2.2. Synthesis and Characterization of Complexes [CuL_2_Cl]^+^[Cl]^−^ and [CuL_2_Cl_2_]

General procedure for the complex preparation. A solution of the corresponding urea (0.2 mmol) in MeOH (4 mL) was added to the solution of CuCl_2_ 5H_2_O (0.1 mmol) in *i*-PrOH (4 mL). The resulting solution was slowly evaporated (approximately 3–4 days) to the precipitation of target Cu(II) complex as crystalline or powder material, which was collected by filtration and dried in air at 50 °C.

In the case of the crystalline precipitate, after the selection of representative samples for X-ray diffraction studies, the remaining material was powdered and dried.

[Cu(**U1**)_2_Cl]^+^[Cl]^−^. Yield 21 mg (46%), green crystals. FTIR (KBr, cm^–1^): 1631 (ν C=O, amide I), 1594 (Pyridine C_2_N), 1518 (ν_a_ CN_2_, amide III), 1471 (Pyridine C_2_N), 791 (γ C=O), 743 (ν_s_ CN_2_, amide II). Anal. calcd. for C_16_H_22_CuCl_2_N_6_O_2_ C 41.34, H 4.77, N 18.08; found: C 41.12 H 4.81, N 17.84. HRMS (ESI), *m/z* [M–Cl]^+^ calcd for C_16_H_22_CuClN_6_O_2_: 428.0783; found: 428.0765.

[Cu(**U2**)_2_Cl]^+^[Cl]^−^. Yield 27 mg (54%), green crystals. FTIR (KBr, cm^–1^): 1640 (ν C=O, amide I), 1625 (ν C=O, amide I) 1588 (Pyridine C_2_N), 1510 (ν_a_ CN_2_ amide III), 1469 (Pyridine C_2_N), 806 (γ C=O), 730 (ν_s_ CN_2_ amide II), 560 (β C=O). Anal. calcd. for C_18_H_26_CuCl_2_N_6_O_2_: C, 43.86; H, 5.32; N, 17.05; found: C, 43.62; H, 5.03; N, 16.58. HRMS (ESI), *m/z* [M–Cl]^+^ calcd for C_18_H_26_CuClN_6_O_2_: 456.1096; found: 456.1098.

[Cu(**U3**)_2_Cl_2_]. Yield 40 mg (72%), greenish yellow crystals. FTIR (KBr, cm^–1^): 1644 (ν C=O, amide I), 1605 (ν C=O, amide I), 1543 (ν_a_ CN_2_ amide III), 1466 (Pyridine C_2_N), 808 (γ C=O), 737 (ν_s_ CN_2_ amide II), 580 (β C=O). Anal. calcd. for C_16_H_20_CuCl_2_N_8_O_6_ + 2MeOH: C 34.93, H 4.56, N 18.11; found: 34.66, H 4.72, N 18.51. HRMS (ESI), *m/z* [M–Cl]^+^ calcd for C_16_H_20_CuClN_8_O_6_: 518.0485; found: 518.0517.

[Cu(**U4**)_2_Cl]^+^[Cl]^−^. Yield 29 mg (57%), green crystals. FTIR (KBr, cm^–1^): 2238 (ν CN), 1649 (ν C=O, amide I), 1610 (ν C=O, amide I) 1587 (Pyridine C_2_N), 1510 (ν_a_ CN_2_ amide III), 1471 (Pyridine C_2_N), 803 (γ C=O), 740 (ν_s_ CN_2_ amide II), 565 (β C=O). Anal. calcd. for C_18_H_20_CuCl_2_N_8_O_2_ + H_2_O: C 40.57, H 4.16, N 21.03; found: C 40.71, H 4.17, N 21.15. HRMS (ESI), *m/z* [M–Cl]^+^ calcd for C_18_H_20_CuClN_8_O_2_: 478.0688; found: 478.0698.

[Cu(**U5**)_2_Cl]^+^[Cl]^−^. Yield 41 mg (65%), green crystals. FTIR (KBr, cm^–1^): 1642 (ν C=O, amide I), 1629 (ν C=O, amide I), 1578 (Pyridine C_2_N), 1530 (ν_a_ CN_2_ amide III), 1461 (Pyridine C_2_N), 802 (γ C=O), 740 (ν_s_ CN_2_ amide II). Anal. calcd. for C_22_H_26_CuCl_2_N_10_O_4_ + 0.33*i*-PrOH: C 42.57, H 4.45, N 21.58; found: C 42.51, H 4.11, N 21.33. HRMS (ESI), *m/z* [M–Cl]^+^ calcd for C_22_H_26_CuClN_10_O_4_: 592.1118; found: 592.1141.

[Cu(**U6**)_2_Cl]^+^[Cl]^−^. Yield 36 mg (58%), green crystals. FTIR (KBr, cm^–1^): 1645 (ν C=O, amide I), 1633 (ν C=O, amide I), 1533 (ν_a_ CN_2_ amide III), 805 (γ C=O), 737 (ν_s_ CN_2_ amide II). Anal. calcd. for C_22_H_26_CuCl_2_N_10_O_4_ + H_2_O: C 40.84, H 4.36, N 21.65; found: C 40.38, H 3.87, N 21.21. HRMS (ESI), *m/z* [M–Cl]^+^ calcd for C_22_H_26_CuClN_10_O_4_: 592.1118; found: 592.1138.

[Cu(**U7**)_2_Cl]^+^[Cl]^−^. Yield 27 mg (52%), green crystals. FTIR (KBr, cm^–1^): 1629 (ν C=O, amide I), 1605 (ν C=O, amide I), 1590 (Pyridine C_2_N), 1517 (ν_a_ CN_2_ amide III), 1485 (Pyridine C_2_N), 795 (γ C=O), 745 (ν_s_ CN_2_ amide II), 540 (β C=O). Anal. calcd. for C_20_H_26_CuCl_2_N_6_O_2_: C 46.47, H 5.07, N 16.26; found: C 46.54, H 5.06, N 16.17. HRMS (ESI), *m/z* [M–Cl]^+^ calcd for C_20_H_26_CuClN_6_O_2_: 480.1096; found: 480.1120.

[Cu(**U8**)_2_Cl]^+^[Cl]^−^. Yield 21 mg (39%), green crystals. FTIR (KBr, cm^–1^): 1624 (ν C=O, amide I), 1602 (ν C=O, amide I), 1602 (ν C=O, amide I), 1586 (Pyridine C_2_N), 1517 (ν_a_ CN_2_ amide III), 1455 (Pyridine C_2_N), 792 (γ C=O), 742 (ν_s_ CN_2_ amide II), 554 (β C=O). Anal. calcd. for C_22_H_30_CuCl_2_N_6_O_2_: C 48.49, H 5.55, N 15.42; found: 48.21 H 5.77, N 15.36. HRMS (ESI), *m/z* [M–Cl]^+^ calcd for C_22_H_30_CuClN_6_O_2_: 508.1409; found: 508.1405.

[Cu(**U9**)_2_Cl]^+^[Cl]^−^. Yield 37 mg (64%), green crystals. FTIR (KBr, cm^–1^): 1640 (ν C=O, amide I), 1617 (ν C=O, amide I), 1593 (Pyridine C_2_N), 1526 (ν_a_ CN_2_ amide III), 1456 (Pyridine C_2_N), 744 (ν_s_ CN_2_ amide II). Anal. calcd. for C_24_H_34_CuCl_2_N_6_O_2_ + 0.5 H_2_O: C 49.53, H 6.06, N 14.44; found: C 49.88, H 5.71, N 14.16. HRMS (ESI), *m/z* [M–Cl]^+^ calcd for C_24_H_34_CuClN_6_O_2_: 536.1722; found: 536.1737.

[Cu(**U10**)_2_Cl]^+^[Cl]^−^. Yield 31 mg (52%), green crystals. FTIR (KBr, cm^–1^): 1632 (ν C=O, amide I), 1587 (Pyridine C_2_N), 1470 (Pyridine C_2_N), 772 (γ C=O), 743 (ν_s_ CN_2_ amide II), 560 (β C=O). Anal. calcd. for C_24_H_34_CuCl_2_N_6_O_4_: C 47.65, H 5.66, N 13.89; found: C 47.39 H 5.69, N 13.40. HRMS (ESI), *m/z* [M–Cl]^+^ calcd for C_24_H_34_CuClN_6_O_4_: 568.1621; found: 568.1629.

[Cu(**U11**)_2_Cl_2_]. Yield 39 mg (61%), greenish yellow crystals. FTIR (KBr, cm^–1^): 1637 (ν C=O, amide I), 1603 (ν C=O, amide I), 1550 (Pyridine C_2_N), 1522 (ν_a_ CN_2_ amide III), 1448 (Pyridine C_2_N), 810 (γ C=O), 743 (ν_s_ CN_2_ amide II), 548 (β C=O). Anal. calcd. for C_22_H_28_CuCl_2_N_8_O_6_: C 41.62, H 4.44, N 17.65; found: C 41.40, H 4.48, N 17.29. HRMS (ESI), *m/z* [M–Cl]^+^ calcd for C_22_H_28_CuClN_8_O_6_: 598.1111; found: 598.1119.

[Cu(**U12**)_2_Cl]^+^[Cl]^−^. Yield 36 mg (63%), green crystals. FTIR (KBr, cm^–1^): 1641 (ν C=O, amide I), 1601 (ν C=O, amide I), 1580 (Pyridine C_2_N), 1520 (ν_a_ CN_2_ amide III), 1466 (Pyridine C_2_N), 780 (γ C=O), 750 (ν_s_ CN_2_ amide II), 582 (β C=O). Anal. calcd. for C_22_H_30_CuC_l2_N_6_O_4_: C 45.80, H 5.24, N 14.57; found: C 45.39, H 5.06, N 14.83. HRMS (ESI), *m/z* [M–Cl]^+^ calcd for C_22_H_30_CuClN_6_O_4_: 540.1308; found: 540.1327.

### 2.3. Crystallography

X-ray diffraction data were collected at a SuperNova diffractometer using Cu-Kα (λ = 0.154184 nm) radiation. The structures have been solved with the ShelXT [39] structure solution program using Intrinsic Phasing and refined with the ShelXL [40] refinement package incorporated in the OLEX2 program package [41] using least squares minimization. The carbon-bound H atoms were placed in calculated positions. Empirical absorption correction was applied in CrysAlisPro [42] program complex using spherical harmonics, implemented in SCALE3 ABSPACK scaling algorithm. X-ray crystallographic data and structural refinement parameters are summarized in Tables S1–S3. Supplementary crystallographic data have been deposited at Cambridge Crystallographic Data Centre (CCDC 2104013, 2104015–2104019, 2104021, 2104091) and can be obtained free of charge via www.ccdc.cam.ac.uk/data_request/cif (accessed on 24 August 2021).

### 2.4. Cell Culture

A549, NCI-H460, NCI-H1975 lung cancer cells and WI-26 VA4 lung epithelial-like cells were purchased from the ATCC. A549 cells were maintained in F12-K (Corning, NY, USA) supplemented with 10% fetal bovine serum (Fetal Bovine Serum, qualified, Australia; Gibco, Loughborough, UK), penicillin (100 UI mL^−1^), streptomycin (100 µg mL^−1^), and GlutaMax (1.9 mM, Gibco, UK). NCI-H460 and NCI-H1975 cells were maintained in RPMI-1640 (ATCC modification) media (Gibco, Loughborough, UK) supplemented with 10% fetal bovine serum (Fetal Bovine Serum, qualified, Australia; Gibco, Loughborough, UK), penicillin (100 UI mL^−1^), streptomycin (100 µg mL^−1^), and GlutaMax (2 mM, Gibco, UK). WI-26 VA4 cells were maintained in Advanced MEM (Gibco, Loughborough, UK) supplemented with 5% fetal bovine serum (Fetal Bovine Serum, qualified, Australia, Gibco, UK), penicillin (100 UI mL^−1^), streptomycin (100 µg mL^−1^), and GlutaMax (1.87 mM, Gibco, Loughborough, UK). All cells line cultivation under a humidified atmosphere of 95% air/5% CO_2_ at 37 °C. Subconfluent monolayers, in the log growth phase, were harvested by a brief treatment with TrypLE Express solution (Gibco, Loughborough, UK) in phosphate buffered saline (PBS, Capricorn Scientific, Germany) and washed three times in serum-free PBS. The number of viable cells was determined by trypan blue exclusion.

### 2.5. MTT Assay 

The effects of the synthesized compounds on cell viability were determined using the MTT colorimetric test. All examined cells were diluted with the growth medium to 3.5 × 10^4^ cells per mL and the aliquots (7 × 10^3^ cells per 200 μL) were placed in individual wells in 96-well plates (Eppendorf, Hamburg, Germany) and incubated for 24 h. The next day, the cells were treated with synthesized compounds separately in concentration 10 and 100 μM (or 200.0 μM concentration and diluted at various concentrations for determination of IC_50_) and incubated for 72 h at 37 °C in 5% CO_2_ atmosphere. Each compound was tested in triplicate. After incubation, the cells were treated with 40 μL MTT solution (3-(4,5-dimethylthiazol-2-yl)-2,5-diphenyltetrazolium bromide, 5 mg mL^−1^ in PBS) and incubated for 4 h. After additional 4-h incubation, the medium with MTT was removed and DMSO (150 μL) was added to dissolve the formazan crystals. The plates were shaken for 10 min. The optical density of each well was determined at 560 nm using GloMax Multi+ (Promega, Madison, WI, USA) microplate reader. Each of the tested compounds was evaluated for cytotoxicity in three separate experiments. All stock solutions for biological evaluations were prepared via dissolving synthesized compounds in DMSO.

### 2.6. Apoptosis Assay

For the detection of apoptosis, the cells were placed at 6-well culture plates (Eppendorf, Germany) and allowed to grow overnight. After the cells reached subconfluency, the medium was replaced with tested compounds (25 or 50 μM). The exposed cells were placed at 37 °C in a 5% CO_2_ incubator for 24 or 48 h. The cultured cells were washed twice with PBS and resuspended in 1× binding buffer (AnnexinV-FITC kit, Invitrogen, Waltham, MA, USA) at a concentration 1 × 10^6^ mL^−1^. Annexin FITC (5 μL) and propidium iodide (PI, 2 μL) were added to 100 μL of the cell suspension and incubated for 15 min at room temperature (25 °C) in the dark. After incubation, 400 μL of 1× binding buffer was added to each tube and the stained cells were analyzed within 1 h using CytoFlex (Beckman Coulter, Brea, CA, USA) and CytExpert 2.1 program. Since the AnnexinV-FITC staining precedes the loss of membrane integrity that accompanies the later stage identified by PI, Annexin FITC positive, PI negative indicates early apoptosis, while the viable cells are Annexin V FITC negative, PI negative. The cells that are in late apoptosis or dead are both Annexin V FITC and PI positive.

### 2.7. Cell Cycle Assay

For the cell cycle analysis, the cells (1 × 10^6^ per well) were placed at 6-well culture plates (Eppendorf, Germany) and allowed to grow overnight. After the medium was replaced with solution tested compounds (10, 20, or 35 μM). The exposed cells were placed at 37 °C in a 5% CO_2_ incubator for 48 h. After the completion of incubations, cells were harvested by trypsinization, washed with PBS, centrifuged, fixed with 70% ethanol and incubated for 7 h at –20 °C. An aliquot of the pellet (200 µL) was washed with PBS, stained with 200 µL of the Guava Cell Cycle Reagent (Merck, Kenilworth, NJ, USA), and incubated for 30 min at room temperature in the dark. The cell cycle distribution was analyzed by flow cytometry on a CytoFlex (Beckham Coulter, Brea, CA, USA) and CytExpert 2.1 program.

## 3. Results

### 3.1. Chemistry

**Scheme 1:**
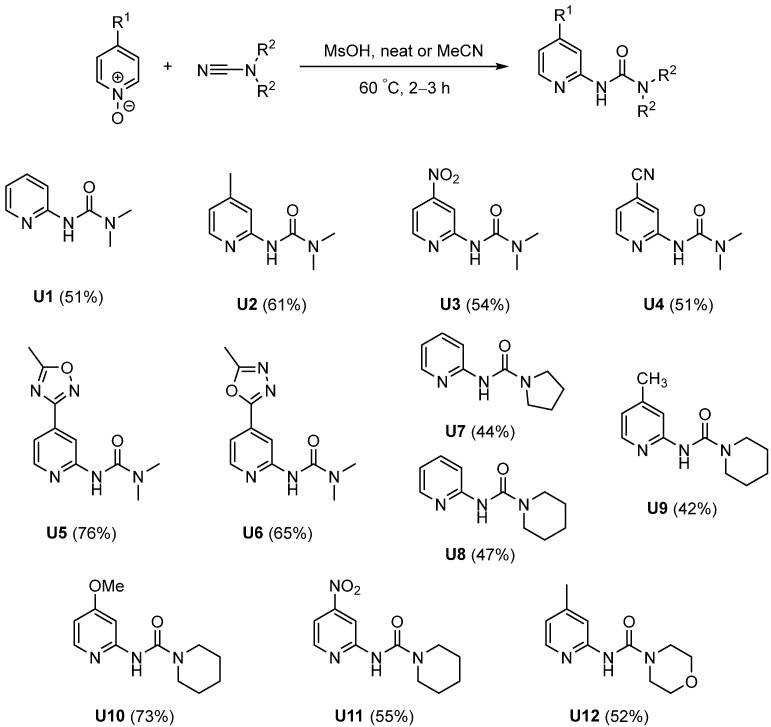
Synthesis of **U1–12** via acid-catalyzed reaction of pyridine-*N*-oxides with dialkylcyanamides.

All 2-pyridyl ureas (**U1–12**) were synthesized via the acid-catalyzed reaction of corresponding *N*-oxides with dialkylcyanamides as described in our previous works (Scheme 1) [33,34,37]. Briefly, the starting materials were mixed and heated in the presence of methanesulfonic acid (MsOH) in acetonitrile or under solvent-free conditions (depending of the reactivity and solubility of starting *N*-oxide). The yields of the desired urea derivatives **U1–12** ranged between 42% and 76%.

**Scheme 2:**
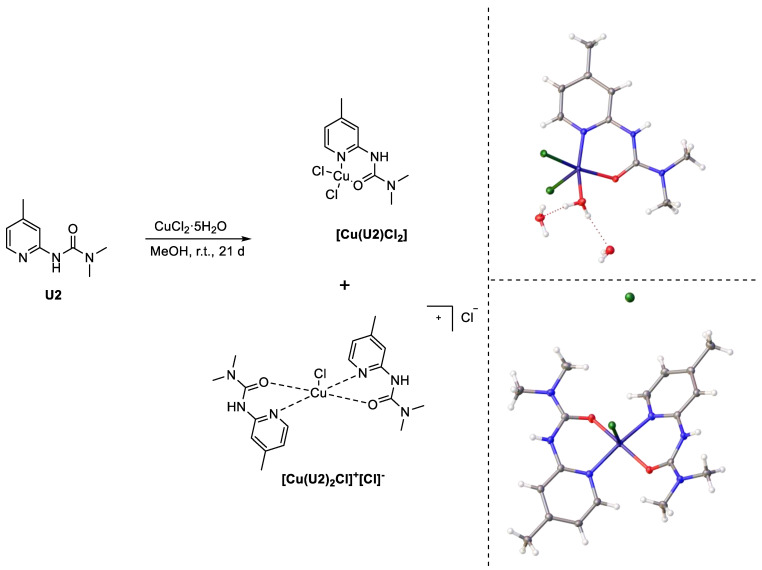
Reaction of **U2** with CuCl_2_ in methanol solution and X-ray structures of obtained mono- and bis-ligand Cu(II) complexes.

This was followed by the preparation of Cu(II) complexes incorporating the obtained 2-pyridyl ureas as ligands. Initially, we attempted to generate the target complexes using traditional approaches reported in the literature procedure [15]. To this end, ethanol solutions of a model ligand (**U2**) and Cu(II) chloride (Scheme 2) were mixed in a 2:1 ratio and then slowly evaporated yielding two types of green crystals. Single-crystal X-ray diffraction analysis revealed that the obtained product contained a mixture of mono-ligand ([CuLCl_2_]) and bis-ligand ([CuL_2_Cl]^+^[Cl]^−^) complexes. The X-ray structures of these compounds are presented in Scheme 2. 

To obtain individual bis-ligand complexes, it was necessary to change the procedure so that the target complexes precipitated from the reaction mixture. The substitution of methanol with ethanol was unsuccessful. It was only the complex of NO_2_-substituted urea derivative ([Cu(**U3**)_2_Cl_2_]) that precipitated from the reaction mixture to give a pure bis-ligand complex in a good yield (72%). All the other products were highly soluble in this solvent. To overcome the product solubility issue, isopropyl alcohol was used as a cosolvent. This helped to reduce the solubility of the target bis-ligand Cu(II) complexes ([CuL_2_Cl_2_]) in the reaction solution, while retaining good solubility of the uncomplexed ligands, CuCl_2_, as well as the expected mono-ligand byproducts. By using these modified conditions, we additionally prepared 11 previously unreported bis-ligand Cu(II) complexes incorporating **U1**, **U2**, and **U4**–**12** derivatives, and the yields comprised 39–65% (Table 1).

All obtained compounds were characterized by elemental analysis, HRMS (electrospray ionization), and IR spectroscopy. *C*, *H*, and *N* elemental analysis confirmed the assumed structures and demonstrated that most of the complexes were obtained as solvates and hydrates. HRMS spectra of all complexes displayed peaks corresponding to ions [CuL_2_Cl]^+^ with characteristic isotopic distribution. 

Complete assignment of vibrational band in IR spectra for these complexes is rather a difficult task both because of the total number of vibrations (more than 100) and because of the strong conjugation of ligand bonds resulting in the significant coupling of vibrational modes. Therefore, we have decided to restrict our analysis to the identification of the principal vibrational modes in the experimental spectra. According to the literature data [43], C=O stretching vibration (amide I mode) should be manifested in the IR spectra of substituted ureas as the strong band at 1676 ± 40 cm^−1^. Based on that analysis we have assigned strong bands at 1641–1694 cm^−1^ as C=O stretching modes of urea moiety in the neat ligands. Upon the coordination to metal ion this band should move to lower frequency, therefore the pairs of bands at 1600–1649 cm^−1^ in vibrational spectra of complexes have been assigned as carbonyl bands, highest one corresponding to out-of-phase and lower—to in-phase vibration of coordinated ligands (no lower frequency band was observed in [Cu(**U1**)_2_Cl]^+^[Cl]^−^ complex because of the centrosymmetric structure). Amide III (ν_a_ CN_2_ stretching) band is usually observed as another strong feature in IR spectra at 1570 ± 60 cm^−1^. We have assigned to this mode strong bands observed at 1522–1533 cm^−1^ for neat ligands. Upon coordination these bands downshift on 15–20 cm^−1^ (except [Cu(**U1**)_2_Cl]^+^[Cl]^−^ where probably out-of-phase ν_a_ CN_2_ mode was observed in IR experimental spectra). Amide II (ν_s_ CN_2_ stretching) band was found as weak to medium vibrational feature at 728–750 cm^−1^ only slightly affected by coordination. CO out-of-plane deformation (γ CO) was observed as medium to strong band at 780–810 cm^−1^ and in-plane CO deformation (β CO) at 550–590 cm^−1^. As to pyridine ring vibrations, according to the data of [44,45,46], CN stretchings contribute mostly to the bands around 1580–1590 and 1455–1480 cm^−1^. Some up-shift of these bands upon coordination is probably due to the significant change in their coupling with other ring vibrations of pyridine moiety (according to potential energy distribution calculations [45] contribution of CN stretching into vibrational modes of pyridine does not exceed 45%). As to the Cu–Cl bands, they should lie below 400 cm^−1^ and were not observed in experimental spectra.

Finally, X-ray diffraction analysis performed for most complexes, verified the proposed structures as outlined in Table 1.

**Figure 1:**
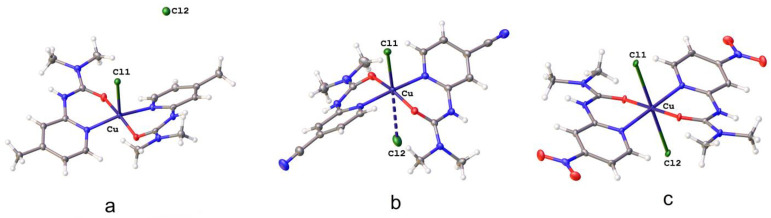
Three types of the obtained Cu(II) complexes as illustrated by X-ray structures of compounds [Cu(**U2**)_2_Cl]^+^[Cl]^−^ (**a**), [Cu(**U4**)_2_Cl]^+^[Cl]^−^ (**b**), and [Cu(**U3**)_2_Cl_2_] (**c**).

The analysis of the X-ray data revealed several structural kinds of the obtained complexes (Figure 1a–c and Table 2). First of all, well pronounced Jahn-Teller effect is observed in all the structures under consideration. That is the common feature of copper(II) complexes with d^9^ configuration of central atom. The presence of strong electron-withdrawing substituent, such as nitro-group, in ligand moiety decreases the nucleophilicity of the pyridine ring and hence both chloride ligands remain in inner coordination sphere resulting in neutral hexacoordinated complex [Cu(**U3**)_2_Cl_2_]. Its structure can be described as tetragonal bipyramidal with chloride ligands in axial positions. Cu–Cl distance is 2.6502 Å that is close to axial interatomic separation in other Cu–Cl complexes with 4 + 2 coordination ca. CsCuCl_3_ (2.73 Å [47]).

Decrease in electron-withdrawing ability of substituents increase the degree of Jahn-Teller distortion leading at first to an intermediate-type complex [Cu(U**4**)_2_Cl]^+^[Cl]^−^ that contains both bridging chloride ligand with Cu–Cl bond distance 2.5614 Å and semi-coordinated to metal center chloride ligand at 3.0220 Å. The last distance is close to that observed in layered copper chlorocomplexes with the trivial bonds in square planar arrangement of copper ion and relatively weak contacts between copper ion and chloride ligands from the neighboring layer (2.96, 3.03 and 3.05 Å for CuCl_2_ [48], KCuCl_3_ [49], and NH_4_CuCl_3_ [49] respectively). This non-equivalency of two ligands in axial positions of distorted octahedral copper complexes is the common case in copper(II) coordination chemistry as it was shown by I. Persson based on EXAFS study of copper complexes in solids and solutions [50].

Further decrease in electron-withdrawing ability of substituents led to the formation of penta-coordinated complexes where only one chloride ligand is coordinated to the metal ion. It is well-known that two kinds of coordination polyhedron can be realized in complexes with coordination number 5. Addison [51] suggested to use τ factor to distinguish between these two opportunities:

τ = (α − β)/60°,

where α is the angle between axial bonds and β is the angle between equatorial bonds. If τ < 0.5, the environment is better described as tetragonal pyramidal, otherwise—as trigonal bipyramidal. Based on this criteria [Cu(**U2**)_2_Cl]^+^[Cl]^−^ and [Cu(**U2**)Cl_2_(H_2_O)] complexes are better described as tetragonal pyramidal that is the next step of Jahn-Teller distortion of octahedral complexes after 4 + 2 and 4 + 1 + 1 geometries. The significantly longer Cu–Cl distances, compared to other penta-coordinated complexes studied (2.525–2.561 Å for apical Cu–Cl bonds vs. 2.27–2.30 Å for equatorial ones), confirm the conclusion about different geometries of penta-coordinated complexes under consideration. The formation of trigonal bipyramidal complexes in other systems is probably due to the steric effects caused by more bulky ligands (inter-ligand repulsion in trigonal bipyramidal complexes is less than in tetragonal pyramidal ones).

Thus, the structural data on the synthesized pyridyl urea-based Cu(II) complexes is in line with previously reported Cu(II) coordination compounds incorporating *N,O*-bidentate ligands [15,21,52,53,54,55].

**Table 2:** Geometry parameters for some of the obtained Cu(II) complexes.

Complex	Cu–Cl1, Å	Cu–Cl2, Å ^1^	Cu–N1, Å	Cu–N4, Å	Cu–O1, Å	Cu–O2, Å
[Cu(**U1**)_2_Cl]^+^[Cl]^−^	2.3055(6)	6.1200(4)	1.9807(13)	1.9807(13)	2.0177(11)	2.0177(11)
[Cu(**U2**)_2_Cl]^+^[Cl]^−^	2.5608(5)	6.7765(6)	2.0048(15)	2.0061(15)	1.9300(13)	1.9270(13)
[Cu(**U3**)_2_Cl_2_]	2.6502(4)	2.6502(4)	2.0047(14)	2.0047(14)	1.9665(13)	1.9665(13)
[Cu(**U4**)_2_Cl]^+^[Cl]^−^	2.5614(4)	3.0220(6) ^1^	1.9907(13)	1.9860(14)	1.9549(12)	1.9518(12)
[Cu(**U7**)_2_Cl]^+^[Cl]^−^	2.2749(5)	6.2742(5)	1.9884(18)	1.9919(18)	2.1184(14)	2.0106(14)
[Cu(**U9**)_2_Cl]^+^[Cl]^−^	2.2827(5)	6.3767(7)	1.9715(15)	1.9768(15)	1.9850(12)	2.1131(12)
[Cu(**U12**)_2_Cl]^+^[Cl]^−^	2.2971(7)	6.5641(8)	1.990(2)	1.986(2)	2.0780(18)	1.9833(18)

^1^ ΣR_vdW_ (Cu + Cl) = 3.15 Å [56].

The measurement of the molar conductivity in a solution in DMSO showed that the complexes of all these three types dissociate upon dissolution in almost the same way, demonstrating the values characteristic of the incomplete dissociation of 1:1 electrolytes (Table 3) [57].

From the point of view of practical use, an important issue is the thermal stability of the obtained complexes. We conducted a thermogravimetric study and showed that these compounds are quite stable with respect to heating (see ESI, Figures S9–S20). For most of the complexes, noticeable destruction began only at temperatures above 200 °C; for several species, this temperature decreased to 130–150 °C. This indicates not only the thermal stability of the complexes, but also the fact that coordination to the copper metal center stabilizes the ligands. The original ureas themselves begin to decompose already at temperatures slightly above 100 °C [37].

### 3.2. Cell Experiments

Given the prominent interest to the novel metal-based drugs, we have evaluated anticancer potential of the newly synthesized pyridyl urea Cu(II) complexes. To do so, we tested the obtained compounds against the human lung cancer cells, including EGFR-mutant and non-mutant cell lines. Lung cancer is the leading cause of cancer-related mortality worldwide, with an overall five-year survival rate of 15% [58]. Drug-resistant phenotypes of lung malignancies show poor response to the conventional and targeted therapeutic agents, which is especially true for the EGFR-mutant cancers [59].

In the first step we have performed standard MTT test [60] of the obtained ligands against A549 human lung carcinoma cells. The test revealed that non-complexed ligands displayed no noticeable antiproliferative activity against the cancer cells at concentrations of 10 and 100 µM (Table 4).

In turn, Cu(II)-complexes were evaluated for their anticancer properties against A549 cells, as well as NCI-H460 non-small cell lung carcinoma cells and EGFR-mutant NCI-H1975 non-small cell lung carcinoma cells. Additionally, we used non-cancerous lung fibroblast WI-26 VA4 to control non-specific cytotoxicity (Table 5).

As can be concluded from the Table 4, the majority of the evaluated complex compounds displayed mild antiproliferative activities against A549 cells with IC_50_ values ranging between 88.7 and 114.5 μM. In fact, it were only the piperidine-substituted compounds [Cu(**U9**)_2_Cl]^+^[Cl]^−^ and [Cu(**U10**)_2_Cl]^+^[Cl]^−^ that displayed somewhat higher activities (76.1 and 86.7 μM respectively) along with two nitropyridine derivatives [Cu(**U3**)_2_Cl_2_] and [Cu(**U11**)_2_Cl_2_] exhibiting half-maximal inhibition at 61.4 and 46.01 correspondingly. The same trend was observed against the NCI-H460 cells. Most of the synthesized molecules showed IC_50_ values above 100 μM when tested against this cell line. Meanwhile, [Cu(**U9**)_2_Cl]^+^[Cl]^−^ and [Cu(**U10**)_2_Cl]^+^[Cl]^−^ possessed more pronounced effects with IC_50_′s between 64.0 and 66.4 μM. As in the case of A549, the most profound activities were displayed by the nitropyridine-containing complexes [Cu(**U3**)_2_Cl_2_] (IC_50_ of 77.5 μM) and [Cu(**U11**)_2_Cl_2_] (IC_50_ of 53.6 μM). Encouragingly, the EGFR-mutant NCI-H1975 non-small cell lung carcinoma cell line turned out to be more sensitive to the treatment with the obtained complexes. Thus, the IC_50_ values of compounds [Cu(**U3**)_2_Cl_2_] and [Cu(**U11**)_2_Cl_2_] were found to be 39.6 μM and 33.4 μM, whereas the rest of compounds displayed half-maximal effects in the range of 61.6–70.2 μM. Noteworthy, non-cancerous lung fibroblast WI-26 VA4 cell line was generally less sensitive to all tested molecules, including the most active nitropyridine derivatives. Actually, the IC_50_ values for the [Cu(**U3**)_2_Cl_2_] and [Cu(**U11**)_2_Cl_2_] against WI-26 VA4 amounted to 81.6 μM and 62.4 μM. Therefore, despite the generally modest activities displayed by the evaluated Cu(II) complexes, two nitropyridine-containing compounds showed enhanced potency against the drug-resistant NCI-H1975 cells, with [Cu(**U3**)_2_Cl_2_] being the most selective over the healthy human cells WI-26 VA4. In the light of these facts, this latter compound was studied for its anticancer activity in more detail. 

### 3.3. Cell Cycle Analysis

**Figure 2:**
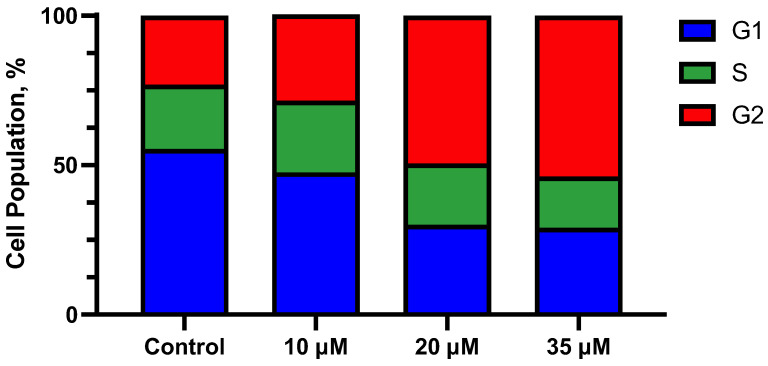
Effects of the compound [Cu(**U3**)_2_Cl_2_] on cell cycle progression in NCI-H1975 non-small cell lung carcinoma cells at concentrations of 10, 20, and 35 μM.

To verify whether the antiproliferative effect of the [Cu(**U3**)_2_Cl_2_] compounds was linked to the cell cycle arrest we analyzed the influence of this complex on the NCI-H1975 cells using flow cytometry. We used three different sub-IC_50_ concentrations in this experiment, namely 10, 20, and 35 μM. The test revealed that [Cu(**U3**)_2_Cl_2_] led to a significant disturbance of the cell cycle in the NCI-H1975 cells, especially at concentrations of 20 and 35 μM. Indeed, treatment of the cells with [Cu(**U3**)_2_Cl_2_] resulted in a profound increase in the cells trapped in the G2 phase. This effect was accompanied by a remarkable drop in the number of cells in the G1 phase, as outlined in Figure 2.

### 3.4. Proapoptotic Activity

**Figure 3:**
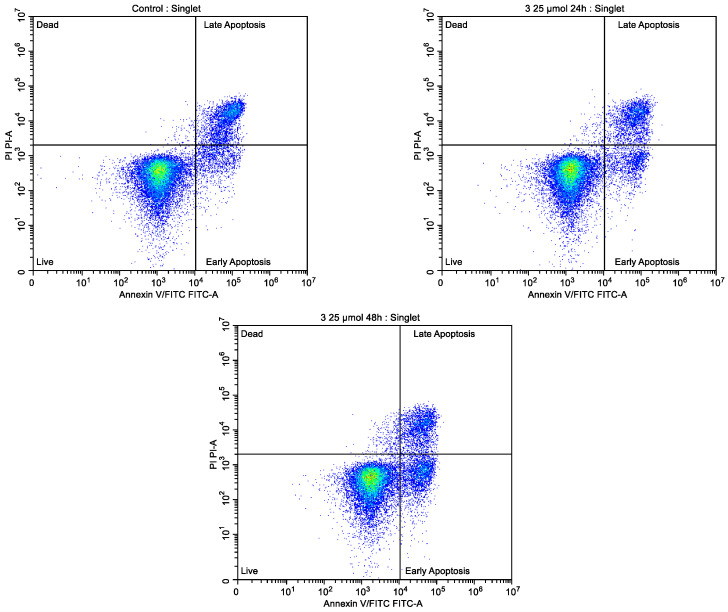

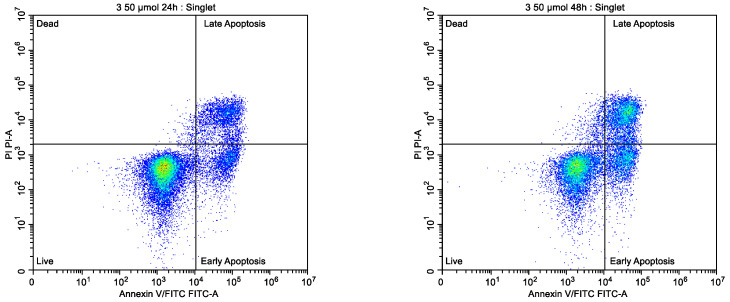
Apoptosis induction caused by different concentrations of the compound [Cu(**U3**)_2_Cl_2_] in NCI-H1975 cell line.

Apoptosis is a physiological process of programmed cell death that is disrupted in malignant cells [61]. Hence, various chemotherapeutic agents with apoptosis induction activity are widely exploited in the context of cancer treatment [62]. Striving to explore the mechanism of cell death underlying the growth inhibitory effect of the synthesized complexes, we have studied the proapoptotic activity of [Cu(**U3**)_2_Cl_2_] in NCI-H1975 cells by means of the Annexin V-FITC/PI double staining assay (Figure 3). 

The obtained morphological data indicated that 48-h incubation of NCI-H1975 cells with 50 μM [Cu(**U3**)_2_Cl_2_] resulted in a noticeable increase in the percent of Annexin VFITC-positive early apoptotic cells from 5.25% to 16.30% which corresponds to three-fold total increase as compared to control. Moreover, the treatment led to the increase in the late apoptotic fraction in cell population from 12.02% to 22.38%. Finally, the percentage of dead cells rose from 0.28% to 2.87%. Thus, at concentration of 50 μM and 48 h incubation time, the proapoptotic effects of the compound [Cu(**U3**)_2_Cl_2_] were rather pronounced. 

## 4. Conclusions

In the present work, we have prepared 12 novel Cu(II) complexes incorporating variously substituted 2-pyridyl ureas as ligands. By means of single-crystal X-ray diffraction analysis, we demonstrated the effect of the substituents’ electronic nature on the binding characteristics of the metal center with chloride ligands. In particular, in the obtained structures Cu–Cl interaction varies from coordination bond (**U3** bearing NO_2_-group in pyridine ring) to dissociation of the ligand into the outer coordination sphere (Me and unsubstituted pyridines). The semi-coordination of chloride ligand to Cu(II) was, also, revealed to the complex of 4-CN substituent in pyridine ring (**U4**).

The synthesized complexes were tested against several lung cancer cell lines (A549, NCI-H460, NCI-H1975) as well as healthy WI-26 VA4 cells. In fact, two nitropyridine-containing complexes (Cu(**U3**)_2_Cl_2_ and Cu(**U11**)_2_Cl_2_) displayed enhanced activity against the drug-resistant NCI-H1975 cells in combination with moderate selectivity toward normal WI-26 VA4 cells. The antiproliferative mechanism of cell death underlying the growth inhibitory effect of the synthesized complexes was studied via additional experiments, including the cell cycle analysis and the apoptosis induction test.

The obtained results highlight the crucial role of the ligand substitution pattern for the antiproliferative properties of Cu(II) 2-pyridyl urea complexes and open room for further structure–activity optimization.

## Figures and Tables

**Table 1:** 2-Pyridyl urea based biligand Cu(II) complexes synthesized in this work.

Entry	Complex	Structure	Yield, %	CCDC Number ^1^
1	[Cu(**U1**)_2_Cl]^+^[Cl]^−^	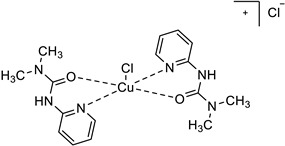	46	2104013
2	[Cu(**U2**)_2_Cl]^+^[Cl]^−^	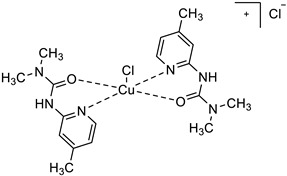	54	2104015
3	[Cu(**U3**)_2_Cl_2_]	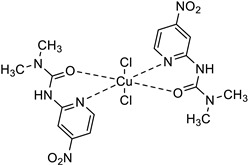	72	2104016
4	[Cu(**U4**)_2_Cl]^+^[Cl]^−^	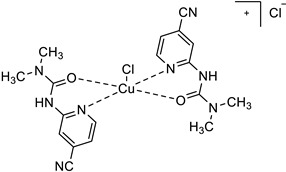	57	2104017
5	[Cu(**U5**)_2_Cl]^+^[Cl]^−^	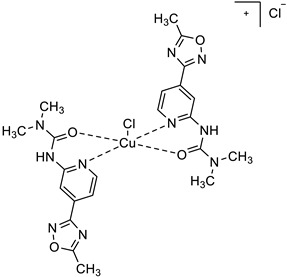	65	
6	[Cu(**U6**)_2_Cl]^+^[Cl]^−^	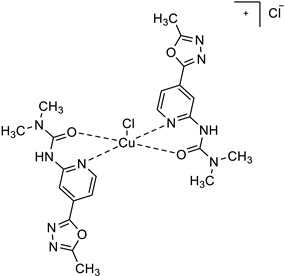	58	
7	[Cu(**U7**)_2_Cl]^+^[Cl]^−^	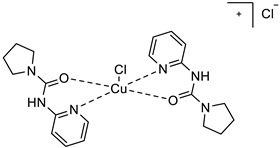	52	2104021
8	[Cu(**U8**)_2_Cl]^+^[Cl]^−^	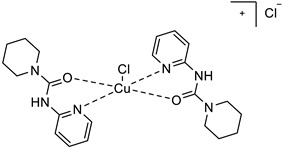	39	
9	[Cu(**U9**)_2_Cl]^+^[Cl]^−^	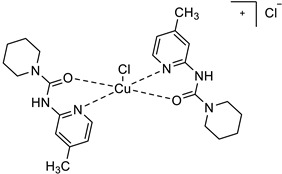	64	2104018
10	[Cu(**U10**)_2_Cl]^+^[Cl]^−^	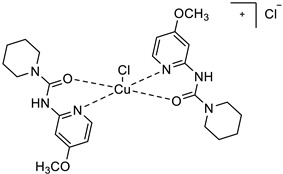	52	
11	[Cu(**U11**)_2_Cl_2_]	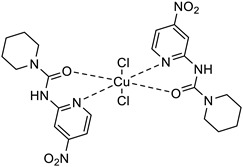	61	
12	[Cu(**U12**)_2_Cl]^+^[Cl]^−^	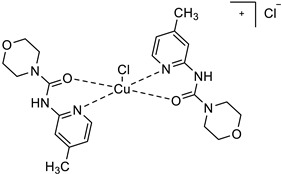	63	2104019

^1^ for structures confirmed by X-ray diffraction analysis.

**Table 3:** Molar conductivity of different type complexes in DMSO solutions (25 °C).

Complex	[Cu(U2)_2_Cl]^+^[Cl]^−^	[Cu(U3)_2_Cl_2_]	[Cu(U4)_2_Cl]^+^[Cl]^−^	[Cu(U11)_2_Cl_2_]
Molar conductivity, Ω^−1^·cm^2^·mol^−1^	24.8	24.1	25.2	21.7

**Table 4:** Results of MTT on A549 cell line for the uncomplexed ligands **U1**–**U12**.

Compound	Cell Viability, %	
	10 µM	100 µM
**U1**	100.5 ± 18.0	86.5 ± 7.5
**U2**	101.5 ± 2.7	98.7 ± 3.8
**U3**	95.5 ± 3.3	91.6 ± 4.3
**U4**	94.2 ± 6.7	89.0 ± 5.1
**U5**	101.2 ± 5.5	90.2 ± 8.7
**U6**	94.5 ± 7.1	89.9 ± 2.4
**U7**	103.3 ± 2.8	99.8 ± 5.7
**U8**	100.3 ± 9.4	97.25 ± 6.1
**U9**	103.2 ± 6.3	98.3 ± 8.1
**U10**	101.8 ± 5.2	99.5 ± 8.1
**U11**	104.8 ± 6.7	94.3 ± 9.0
**U12**	97.4 ± 5.5	85.4 ± 7.8
control	100.0 ± 3.7	

**Table 5:** MTT assay results for the compounds against lung cancer cell lines.

Compound	IC_50_, μM			
	A549	NCI-H460	NCI-H1975	WI-26 VA4
[Cu(**U1**)_2_Cl]^+^[Cl]^−^	106.8 ± 15.1	120.3 ± 18.4	65.3 ± 10.9	108.3 ± 27.5
[Cu(**U2**)_2_Cl]^+^[Cl]^−^	94.5 ± 14.1	121.6 ± 15.3	65.4 ± 8.6	100.8 ± 15.1
[Cu(**U3**)_2_Cl_2_]	61.4 ± 6.52	77.5 ± 6.4	39.6 ± 4.5	81.6 ± 10.1
[Cu(**U4**)_2_Cl]^+^[Cl]^−^	106.5 ± 12.2	118.0 ± 13.4	68.3 ± 8.5	108.8 ± 16.4
[Cu(**U5**)_2_Cl]^+^[Cl]^−^	114.5 ± 14.5	136.3 ± 12.5	71.2 ± 6.9	117.5 ± 24.9
[Cu(**U6**)_2_Cl]^+^[Cl]^−^	88.7 ± 12.7	115.4 ± 26.3	63.8 ± 8.7	104.7 ± 14.1
[Cu(**U7**)_2_Cl]^+^[Cl]^−^	102.0 ± 15.8	102.8 ± 13.1	66.7 ± 7.6	100.2 ± 16.6
[Cu(**U8**)_2_Cl]^+^[Cl]^−^	98.4 ± 11.1	101.6 ± 17.3	64.4 ± 10.5	111.2 ± 19.3
[Cu(**U9**)_2_Cl]^+^[Cl]^−^	76.1 ± 8.86	66.4 ± 5.7	61.6 ± 6.0	112.8 ± 23.5
[Cu(**U10**)_2_Cl]^+^[Cl]^−^	86.7 ± 9.72	64.0 ± 5.7	62.3 ± 7.0	104.2 ± 17.4
[Cu(**U11**)_2_Cl_2_]	46.01 ± 4.98	53.6 ± 4.7	33.4 ± 3.8	62.4 ± 5.6
[Cu(**U12**)_2_Cl]^+^[Cl]^−^	89.3 ± 6.67	128.0 ± 19.5	70.2 ± 4.9	109.3 ± 13.7

## Data Availability

Data are shown in the article and supporting materials. CIFs are openly available in www.ccdc.cam.ac.uk/data_request/cif (accessed on 24 August 2021).

## Supplementary Materials

The following are available online at https://www.mdpi.com/2227-9059/10/2/461/s1 and contains characterization data for ligands and crystallographic information (Tables S1–S3), X-ray structures (Figures S1–S8), and TG/DTG curves for obtained complexes (Figures S9–S20).
