# Supplementary material for: Synthesis, Structure, and Antiproliferative Action of 2-Pyridyl Urea-Based Cu(II) Complexes
Source: Biomedicines. 2022 Feb 16;10(2):461. doi: 10.3390/biomedicines10020461 (PMC8962293; doi:10.3390/biomedicines10020461)
Supplement: Supplementary file 1 [file biomedicines-10-00461-s001.zip › biomedicines-1374713-supplementary.pdf]

Supporting Information

for

Synthesis, Structure and Antiproliferative Action of 2-Pyridyl Urea-Based Cu(II) Complexes

Kirill K. Geyl[1], Sergey V. Baykov[1,]*, Stanislav A. Kalinin[1,2], Alexander S. Bunev[2], Marina A. Troshina[2], Tatiana V. Sharonova[1], Mikhail Yu. Skripkin[1], Svetlana O. Kasatkina[1], Sofia I. Presnukhina[3], Anton A. Shetnev[3], Mikhail Yu. Krasavin[1], Vadim P. Boyarskiy[1]

Address:

[1]Institute of Chemistry, Saint Petersburg State University, Universitetsky pr. 26, Saint Petersburg, 198504, Russian Federation.

[2]Medicinal Chemistry Center, Togliatti State University, Togliatti, 445020, Russian Federation

[3]Pharmaceutical Technology Transfer Center, Yaroslavl State Pedagogical University named after K.D. Ushinskiy, 108 Respublikanskaya St., Yaroslavl, 150000, Russian Federation.

* Corresponding author: s.baykov@spbu.ru

Content

1. Synthesis and characterization of Urea1–12 3
2. Crystallographic information 8
3. TG/DTG data of the synthesized Cu(II) complexes 16
4. References 22

1. Synthesis and characterization of Urea1–12

U1-12 were synthesized from corresponding *N*-oxides according to literature procedures [1,2]. **U1–4** and **U7–8** were described in work [1], **U5** was decribed in work [2], **U6** was decribed in work [3], and **U9–12** were decribed in work [4].

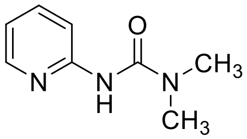

1,1-Dimethyl-3-(pyridin-2-yl)urea (U1) [1]. Yield 84 mg (51%); pale yellow solid; mp 40–42 °C. ^{1}H NMR (400 MHz, $CDCl_3$): δ 8.20 (d, J = 3.6 Hz, 1H), 8.08 (d, J = 8.4 Hz, 1H), 7.71–7.62 (m, 1H), 6.99–6.90 (m, 1H), 3.07 (s, 6H). ^{13}C NMR (101 MHz, $CDCl_3$): δ 154.80, 152.77, 147.27, 137.98, 118.14, 113.04, 36.30.

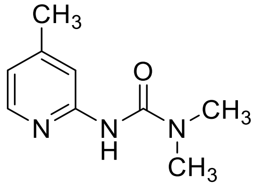

1,1-Dimethyl-3-(4-methylpyridin-2-yl)urea (U2) [1]. Yield 110 mg (61%); pale yellow solid; mp 103–104 °C. ^{1}H NMR (400 MHz, $CDCl_3$): δ 8.06 (d, J = 5.2 Hz, 1H), 7.93 (s, 1H), 7.11 (s, 1H), 6.79 (d, J = 5.2 Hz, 1H), 3.07 (s, 6H), 2.35 (s, 3H). ^{13}C NMR (101 MHz, $CDCl_3$): δ 154.94, 152.82, 149.47, 147.03, 119.57, 113.51, 36.38, 21.37.

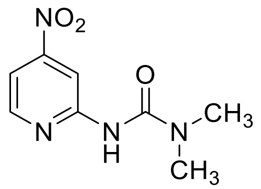

1,1-Dimethyl-3-(4-nitropyridin-2-yl)urea (U3) [1**]**. Yield 114 mg (54%); white solid; mp 124–125 °C. ^{1}H NMR (400 MHz, $CDCl_3$): δ 8.83 (d, J = 1.6 Hz, 1H), 8.42 (d, J = 5.2 Hz, 1H), 7.62 (dd, J = 5.2, 2.0 Hz, 1H), 7.51 (s, 1H), 3.09 (s, 6H). ^{13}C NMR (101 MHz, $CDCl_3$): δ 155.30, 154.97, 154.11, 149.36, 110.68, 106.23, 36.36.

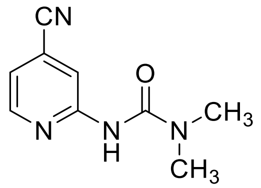

3-(4-Cyanopyridin-2-yl)-1,1-dimethylurea (U4) [1**]**. Yield 97 mg (51%); white solid; mp 98–99 °C. ^{1}H NMR (400 MHz, $CDCl_3$): δ 8.41 (s, 1H), 8.34 (d, J = 5.2 Hz, 1H), 7.34 (s, 1H), 7.14 (d, J = 4.0 Hz, 1H), 3.08 (s, 6H). ^{13}C NMR (101 MHz, $CDCl_3$): δ 154.14, 153.53, 148.59, 121.99, 119.39, 116.70, 115.18, 36.39.

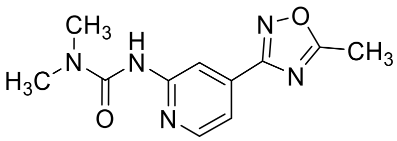

1,1-Dimethyl-3-(4-(5-methyl-1,2,4-oxadiazol-3-yl)pyridin-2-yl)urea (U5) [2**]**. Yield 188 mg (76%); yellow solid; mp 140–142 °C. ^{1}H NMR (400 MHz, $CDCl_3$): δ 8.77 (s, 1H), 8.31 (d, J = 5.2 Hz, 1H), 7.57 (dd, J = 5.2, 1.3 Hz, 1H), 7.54–7.43 (s, 1H), 3.08 (s, 6H), 2.66 (s, 3H). ^{13}C NMR (101 MHz, $CDCl_3$): δ 177.1, 167.1, 154.5, 153.7, 147.7, 136.6, 115.8, 111.4, 36.5, 12.3.

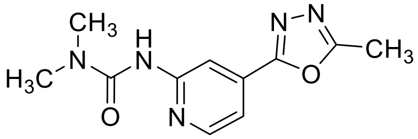

1,1-Dimethyl-3-(4-(5-methyl-1,3,4-oxadiazol-2-yl)pyridin-2-yl)urea (U6) [3**].** Yield 161 mg (65%); yellow solid; mp 193–195 °C. ^{1}H NMR (400 MHz, DMSO-d_6): δ 9.21 (s, 1H), 8.44 (dd, J = 5.2, 0.8 Hz, 1H), 8.42–7.40 (s, 1H), 7.49 (dd, J = 5.2, 1.5 Hz, 1H), 2.98 (s, 6H), 2.62 (s, 3H). ^{13}C NMR (101 MHz, DMSO-d_6): δ 165.3, 163.3, 155.5, 155.3, 149.4, 132.3, 114.2, 109.7, 36.7, 11.1.

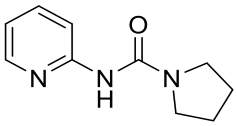

***N-(*Pyridin-2-yl)pyrrolidine-1-carboxamide (U7) [**1]. Yield 84 mg (44%); pale yellow solid; mp 116–117 °C. ^{1}H NMR (400 MHz, CDCl$_3$): δ 8.20–8.16 (m, 1H), 8.11 (dt, J = 8.4, 1.2 Hz, 1 H), 7.67–7.63 (m, 1H), 7.00 (s, 1H), 6.92 (ddd, J = 7.2, 5.2, 1.2 Hz, 1H), 3.52–3.46 (m, 4H), 1.98–1.94 (m, 4H). ^{13}C NMR (101 MHz, CDCl$_3$): δ 153.01, 152.68, 147.30, 137.92, 118.02, 112.85, 45.69, 25.43.

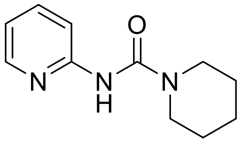

***N-(*Pyridin-2-yl)pyrrolidine-1-carboxamide (U8) [**1**]**. Yield 97 mg (47%); white solid; mp 87–88 °C. ^{1}H NMR (400 MHz, CDCl$_3$): δ 8.24–8.16 (m, 1H), 8.02 (d, J = 8.4 Hz, 1H), 7.64 (s, 1H),

7.28 (s, 1H), 6.93 (d, *J* = 2.4 Hz, 1H), 3.56–3.41 (m, 4H), 1.71–1.57 (m, 6H). ^{13}C NMR (101 MHz, $CDCl_3$): *δ* 153.92, 153.02, 147.44, 138.01, 118.14, 113.26, 45.20, 25.71, 24.37.

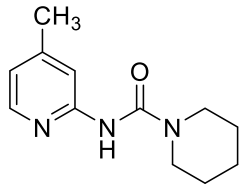

***N*-(Pyridin-2-yl)piperidine-1-carboxamide (U9) [**3**]**. Yield 92 mg (42%); white solid; mp 108–109 °C. ^{1}H NMR (400 MHz, $CDCl_3$): *δ* 8.06 (d, *J* = 5.2 Hz, 1H), 7.90 (s, 1H), 7.15 (s, 1H), 6.78 (d, *J* = 5.2 Hz, 1H), 3.50 (t, *J* = 5.2 Hz, 4H), 2.35 (s, 3H), 1.71–1.62 (m, 6H). ^{13}C NMR (101 MHz, $CDCl_3$): *δ* 154.0, 153.0, 149.4, 147.1, 119.5, 113.7, 45.2, 25.7, 24.5, 21.4.

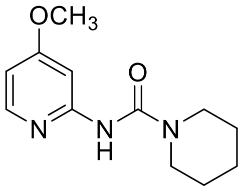

***N*-(4-Methoxypyridin-2-yl)piperidine-1-carboxamide (U10) [**3**]**. Yield 172 mg (73%); white solid; mp 74–75 °C. ^{1}H NMR (400 MHz, $CDCl_3$): *δ* 7.99 (d, *J* = 5.9 Hz, 1H), 7.72 (d, *J* = 2.4 Hz, 1H), 7.25 (s, 1H), 6.51 (dd, *J* = 5.9, 2.4 Hz, 1H), 3.58–3.43 (m, 4H), 1.73–1.55 (m, 6H). ^{13}C NMR (101 MHz, $CDCl_3$): *δ* 167.56, 154.72, 153.92, 147.37, 106.92, 97.16, 55.32, 45.23, 25.71, 24.36.

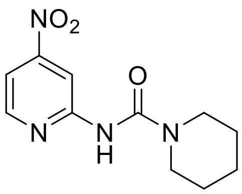

***N*-(4-Nitropyridin-2-yl)piperidine-1-carboxamide (U11) [**3**]**. Yield 138 mg (55%); orange solid; mp 119–121 °C. ^{1}H NMR (400 MHz, $CDCl_3$): δ 8.83 (d, J = 2.0 Hz, 1H), 8.42 (d, J = 5.6 Hz, 1H), 7.79–7.50 (m, 2H), 3.57–3.48 (m, 4H), 1.75–1.59 (m, 6H). ^{13}C NMR (101 MHz, $CDCl_3$): δ 155.42, 155.22, 153.07, 149.13, 110.65, 106.65, 45.31, 25.69, 24.24.

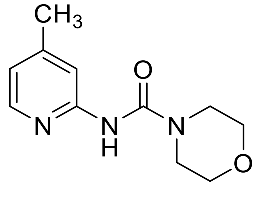

***N*-(4-Methylpyridin-2-yl)morpholine-4-carboxamide (U12) [**3]. Yield 115 mg (52%); white solid; mp 56–58 °C. ^{1}H NMR (400 MHz, $CDCl_3$): δ 8.05 (d, J = 4.6 Hz, 1H), 7.88 (s, 1H), 7.32 (s, 1H), 6.80 (d, J = 4.5 Hz, 1H), 3.84–3.64 (m, 4H), 3.62–3.42 (m, 4H), 2.36 (s, 3H). ^{13}C NMR (101 MHz, $CDCl_3$): δ 154.3, 152.6, 150.1, 146.4, 119.8, 114.1, 66.5, 44.3, 21.5.

2. Crystallographic information

Table S1. Crystal data and structure refinement for $[Cu(\mathbf{U1})_2Cl]^+[Cl]^-$, $[Cu(\mathbf{U2})_2Cl]^+[Cl]^-$ and $[Cu(\mathbf{U3})_2Cl_2]$.

Identification code	$[Cu(\mathbf{U1})_2Cl]^+[Cl]^-$	$[Cu(\mathbf{U2})_2Cl]^+[Cl]^-$	$[Cu(\mathbf{U3})_2Cl_2]$
Datablock	KKG-373	BSC-235	**BSC-136**
CCDC number	2104013	2104015	2104016
Empirical formula	$C_{16}H_{22}Cl_2CuN_6O_2$	$C_{18}H_{34}Cl_2CuN_6O_6$	$C_{16}H_{20}Cl_2CuN_8O_6$
Formula weight	464.83	564.95	554.84
Temperature/K	100(2)	100(2)	100(2)
Crystal system	monoclinic	triclinic	triclinic
Space group	I2/a	P-1	P-1
a/Å	12.7868(6)	7.7544(2)	7.8546(4)
b/Å	12.7469(5)	12.2959(4)	8.3151(5)
c/Å	12.9949(7)	14.3799(5)	8.8607(5)
α/°	90	107.947(3)	80.009(5)
β/°	112.191(6)	99.809(3)	68.652(5)
γ/°	90	96.431(3)	88.264(4)
Volume/$Å^3$	1961.18(18)	1265.36(7)	530.49(6)
Z	4	2	1
ρ_{calc} g/cm^3	1.574	1.483	1.737
μ/mm^{-1}	4.293	3.549	4.262
F(000)	956.0	590.0	283.0
Crystal size/mm^3	0.13 × 0.13 × 0.13	0.17 × 0.14 × 0.11	0.12 × 0.12 × 0.1
Radiation	Cu Kα (λ = 1.54184)	Cu Kα (λ = 1.54184)	Cu Kα (λ = 1.54184)
2Θ range for data collection/°	10.11 to 154.476	6.618 to 152.352	10.81 to 139.862
Index ranges	$-13 \le h \le 15$, $-15 \le k \le 15$, $-16 \le l \le 16$	$-9 \le h \le 9$, $-15 \le k \le 15$, $-17 \le l \le 17$	$-9 \le h \le 8$, $-10 \le k \le 9$, $-10 \le l \le 10$
Reflections collected	9230	23046	4848
Independent reflections	2026 [R_{int} = 0.0332, R_{sigma} = 0.0216]	5210 [R_{int} = 0.0494, R_{sigma} = 0.0326]	2000 [R_{int} = 0.0202, R_{sigma} = 0.0165]
Data/restraints/parameters	2026/0/136	5210/0/320	2000/0/154
Goodness-of-fit on F^2	1.052	1.038	1.096
Final R indexes [I≥2σ (I)]	R_1 = 0.0284, wR_2 = 0.0798	R_1 = 0.0310, wR_2 = 0.0799	R_1 = 0.0262, wR_2 = 0.0713
Final R indexes [all data]	R_1 = 0.0294, wR_2 = 0.0807	R_1 = 0.0366, wR_2 = 0.0839	R_1 = 0.0265, wR_2 = 0.0715
Largest diff. peak/hole / $e \cdot Å^{-3}$	0.36/–0.48	0.39/–0.42	0.33/–0.53

Table S2. Crystal data and structure refinement for $[Cu(\mathbf{U4})_2Cl]^+[Cl]^-$, $[Cu(\mathbf{U7})_2Cl]^+[Cl]^-$ and $[Cu(\mathbf{U9})_2Cl]^+[Cl]^-$.

Identification code	$[Cu(\mathbf{U4})_2Cl]^+[Cl]^-$	$[Cu(\mathbf{U7})_2Cl]^+[Cl]^-$	$[Cu(\mathbf{U9})_2Cl]^+[Cl]^-$
Datablock	KKG-222	KKG-265	KKG-195
CCDC number	2104017	2104021	2104018
Empirical formula	$C_{36}H_{40}Cl_4Cu_2N_{16}O_4$	$C_{20}H_{26}Cl_2CuN_6O_2$	$C_{24}H_{34}Cl_2CuN_6O_2$
Formula weight	1029.72	516.91	573.01
Temperature/K	100(2)	100(2)	100(2)
Crystal system	monoclinic	triclinic	monoclinic
Space group	P2/n	P-1	$P2_1/n$
a/Å	12.48230(10)	7.9567(2)	10.0692(3)
b/Å	13.25080(10)	11.9417(2)	24.5458(7)
c/Å	14.8414(2)	12.4374(2)	10.6384(3)
α/°	90	73.1237(17)	90
β/°	110.6690(10)	74.621(2)	92.502(3)
γ/°	90	88.1870(19)	90
Volume/$Å^3$	2296.77(4)	1089.07(4)	2626.85(14)
Z	2	2	4
ρ_{calc} g/cm^3	1.489	1.576	1.449
μ/mm^{-1}	3.757	3.932	3.316
F(000)	1052.0	534.0	1196.0
Crystal size/mm^3	0.13 × 0.12 × 0.11	0.16 × 0.14 × 0.13	0.2 × 0.11 × 0.1
Radiation	CuKα (λ = 1.54184)	Cu Kα (λ = 1.54184)	Cu Kα (λ = 1.54184)
2Θ range for data collection/°	6.67 to 140.944	7.71 to 140.848	7.202 to 152.238
Index ranges	$-15 \le h \le 10$, $-16 \le k \le 16$, $-18 \le l \le 17$	$-9 \le h \le 9$, $-10 \le k \le 14$, $-15 \le l \le 15$	$-12 \le h \le 12$, $-30 \le k \le 30$, $-13 \le l \le 13$
Reflections collected	18842	10940	17243
Independent reflections	4350 [R_{int} = 0.0357, R_{sigma} = 0.0226]	4154 [R_{int} = 0.0536, R_{sigma} = 0.0519]	5407 [R_{int} = 0.0368, R_{sigma} = 0.0349]
Data/restraints/parameters	4350/0/296	4154/0/280	5407/0/336
Goodness-of-fit on F^2	1.072	1.062	0.971
Final R indexes [I≥2σ (I)]	R_1 = 0.0304, wR_2 = 0.0853	R_1 = 0.0400, wR_2 = 0.1100	R_1 = 0.0312, wR_2 = 0.0785
Final R indexes [all data]	R_1 = 0.0314, wR_2 = 0.0862	R_1 = 0.0432, wR_2 = 0.1125	R_1 = 0.0374, wR_2 = 0.0831
Largest diff. peak/hole / $e·Å^{-3}$	0.49/–0.52	0.36/–0.60	0.61/–0.46

Table S3. Crystal data and structure refinement for [Cu(U1)$_2$Cl]$^+$[Cl]$^-$ and [Cu(U2)Cl$_2$H$_2$O**]**.

Identification code	[Cu(**U12**)$_2$Cl]$^+$[Cl]$^-$	[Cu(**U2**)Cl$_2$H$_2$O]
Datablock	KKG-297	BSC-106
CCDC number	2104019	2104091
Empirical formula	$C_{22}H_{30}Cl_2CuN_6O_4$	$C_9H_{19}Cl_2CuN_3O_4$
Formula weight	576.96	367.71
Temperature/K	100(2)	100(2)
Crystal system	triclinic	triclinic
Space group	P-1	P-1
a/Å	8.8800(4)	7.2201(2)
b/Å	11.2254(4)	9.7425(2)
c/Å	12.9758(4)	11.9473(3)
α/°	81.611(3)	66.272(2)
β/°	81.774(3)	83.676(2)
γ/°	87.818(3)	73.567(2)
Volume/Å^3	1266.25(8)	737.92(3)
Z	2	2
ρ_{calc} g/cm^3	1.513	1.655
μ/mm^{-1}	3.507	5.558
F(000)	598.0	378.0
Crystal size/mm^3	0.15 × 0.13 × 0.11	0.16 × 0.15 × 0.12
Radiation	Cu Kα (λ = 1.54184)	CuKα (λ = 1.54184)
2Θ range for data collection/°	6.954 to 140.818	8.084 to 145.55
Index ranges	–10 ≤ h ≤ 10,<br>–13 ≤ k ≤ 10,<br>–15 ≤ l ≤ 15	–8 ≤ h ≤ 8, -12 ≤ k ≤ 12,<br>–14 ≤ l ≤ 14
Reflections collected	14682	6718
Independent reflections	4778 [R_{int} = 0.0467, R_{sigma} = 0.0430]	2751 [R_{int} = 0.0215, R_{sigma} = 0.0246]
Data/restraints/parameters	4778/0/341	2751/9/199
Goodness-of-fit on F^2	1.066	1.090
Final R indexes [I≥2σ (I)]	R_1 = 0.0450,<br>wR_2 = 0.1222	R_1 = 0.0250,<br>wR_2 = 0.0648
Final R indexes [all data]	R_1 = 0.0494,<br>wR_2 = 0.1258	R_1 = 0.0254,<br>wR_2 = 0.0651
Largest diff. peak/hole / e·Å^{-3}	0.83/–0.81	0.30/–0.39

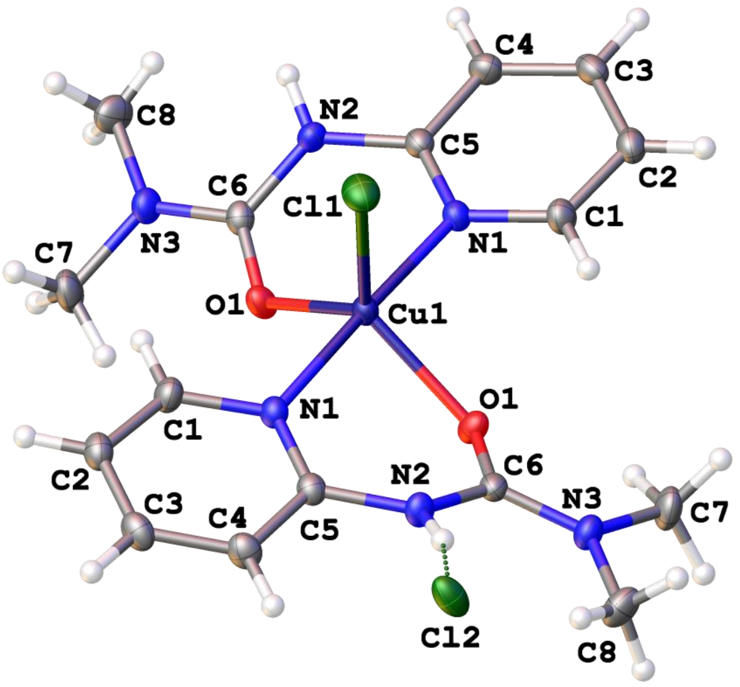

Figure S1. Structure of [Cu(**U1**)$_2$Cl]$^+$[Cl]$^-$.

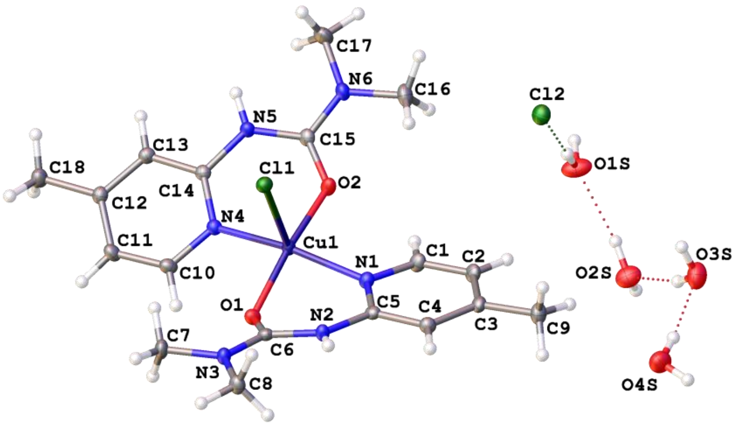

Figure S2. Structure of [Cu(**U2**)$_2$Cl]$^+$[Cl]$^-$.

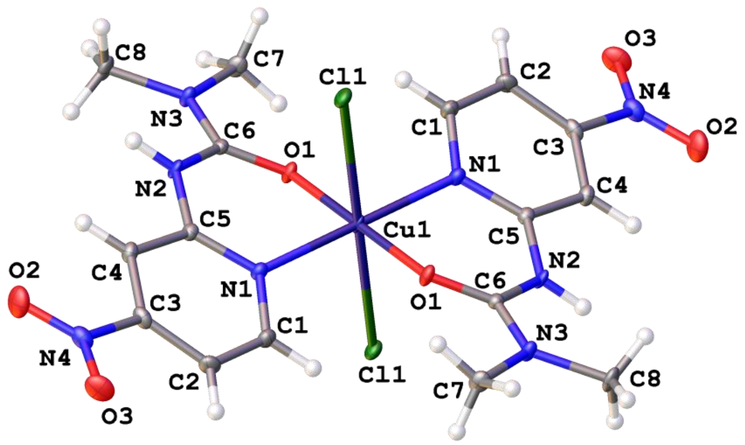

Figure S3. Structure of [Cu(**U3**)$_2$Cl$_2$].

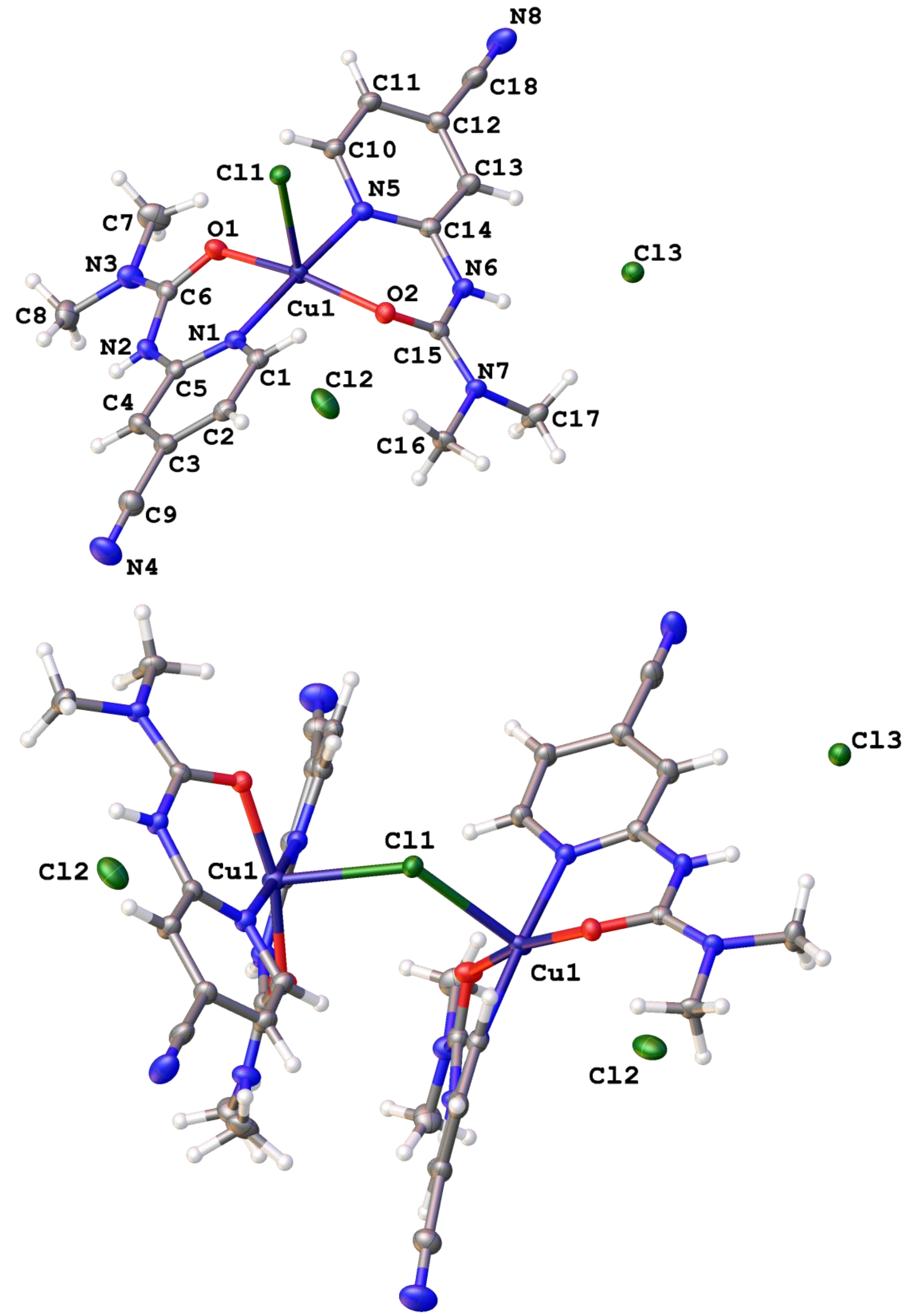

Figure S4. Structure of $[Cu(\mathbf{U4})_2Cl]^+[Cl]^-$.

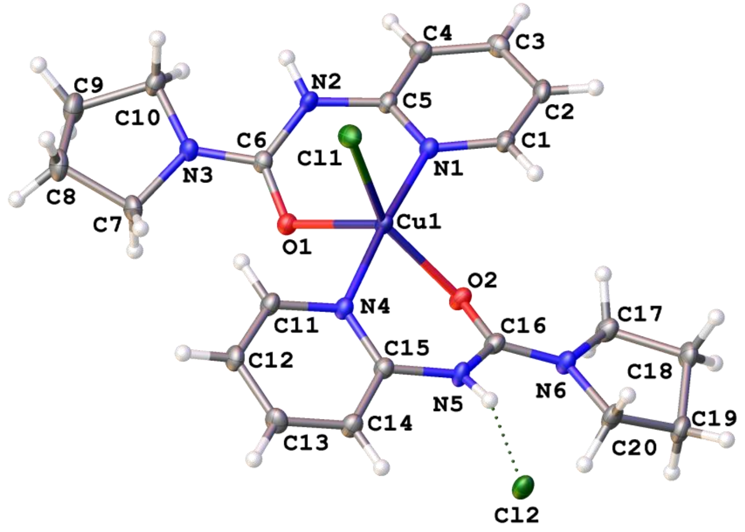

Figure S5. Structure of $[Cu(\mathbf{U7})_2Cl]^+[Cl]^-$.

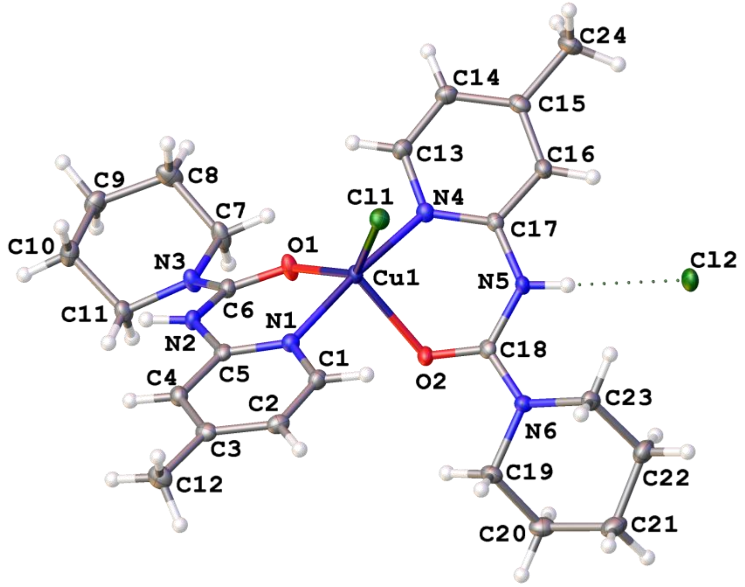

Figure S6. Structure of $[Cu(\mathbf{U9})_2Cl]^+[Cl]^-$.

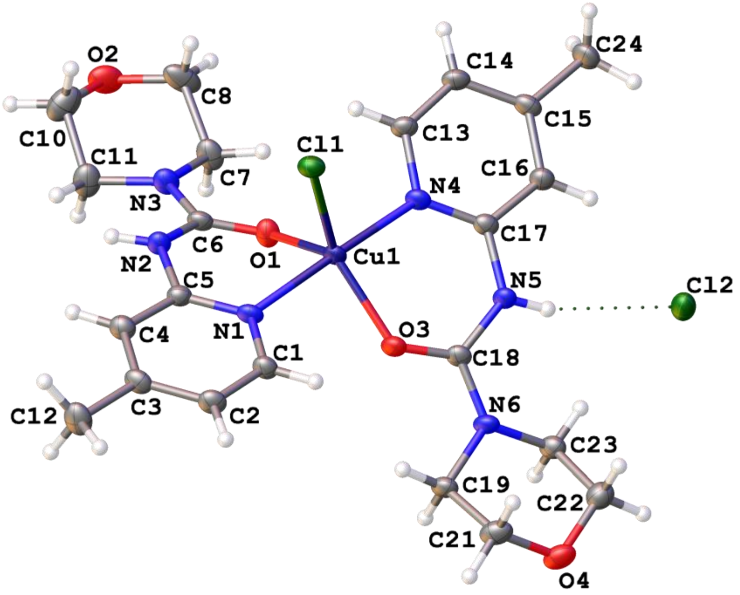

Figure S7. Structure of $[Cu(\mathbf{U12})_2Cl]^+[Cl]^-$.

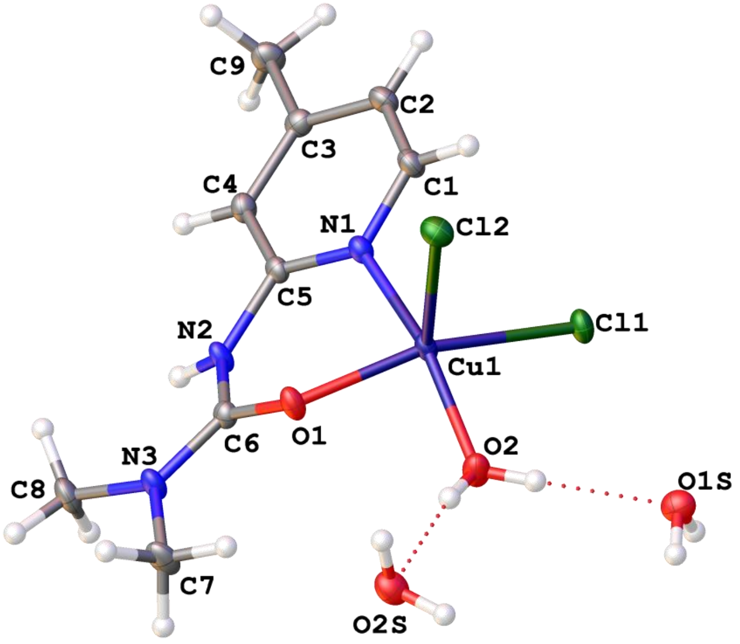

Figure S8. Structure of [Cu(**U2**)Cl_2H2O].

3. TG/DTG data of the synthesized Cu(II) complexes

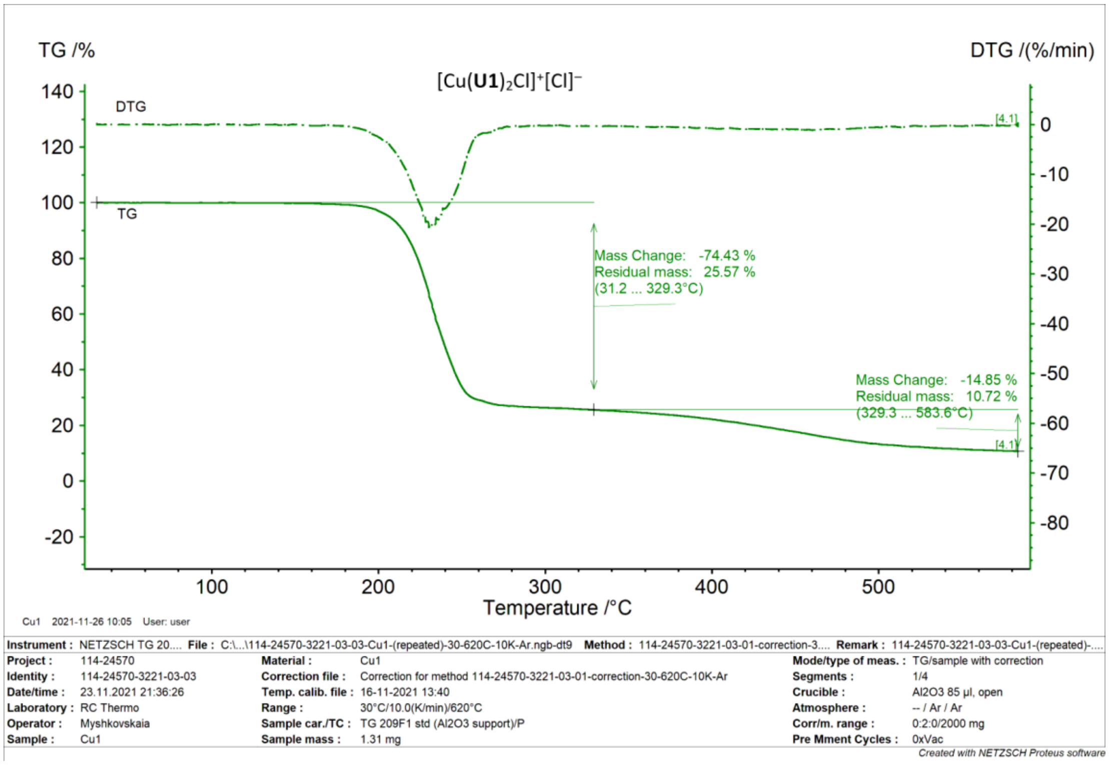

Figure S9. TG/DTG curves of $[Cu(\mathbf{U1})_2Cl]^+[Cl]^-$.

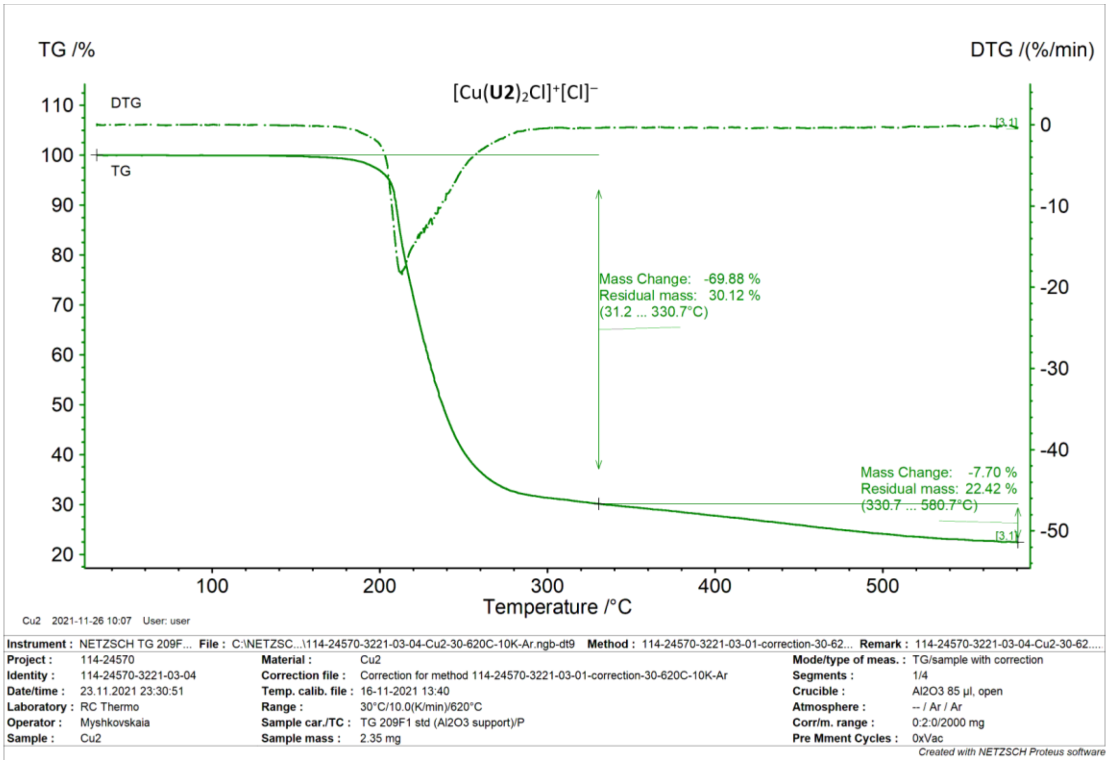

Figure S10. TG/DTG curves of $[Cu(\mathbf{U2})_2Cl]^+[Cl]^-$.

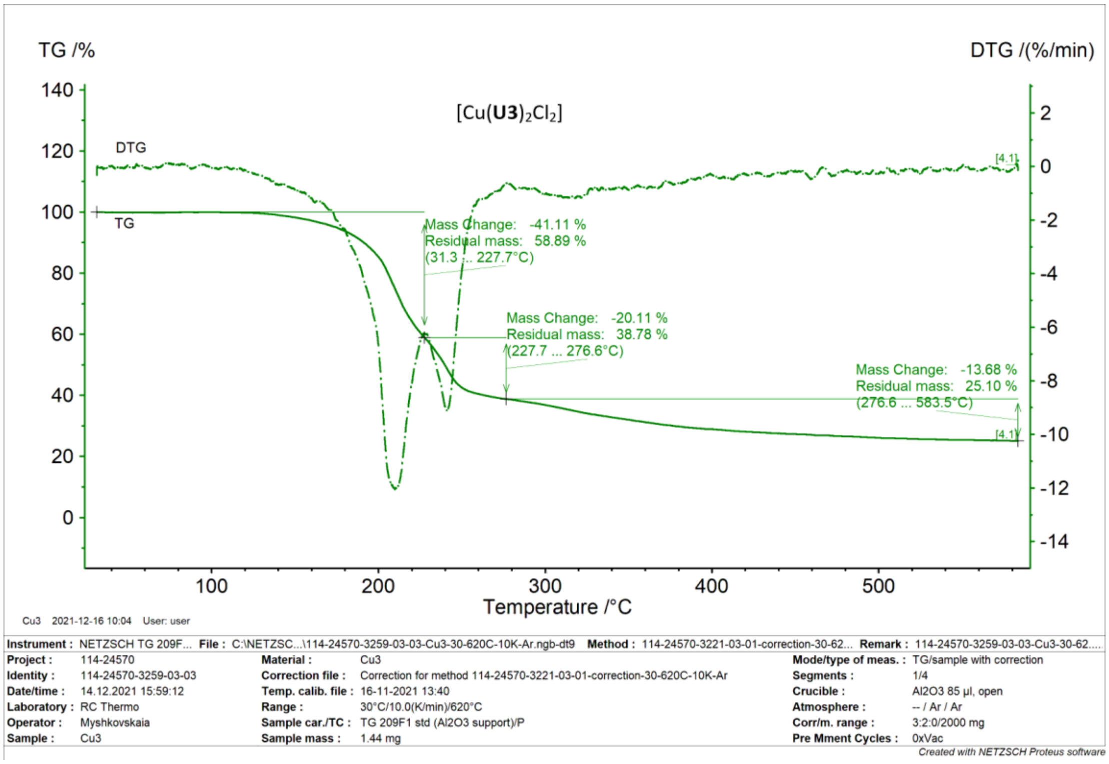

Figure S11. TG/DTG curves of [Cu(**U3**)$_2$Cl$_2$].

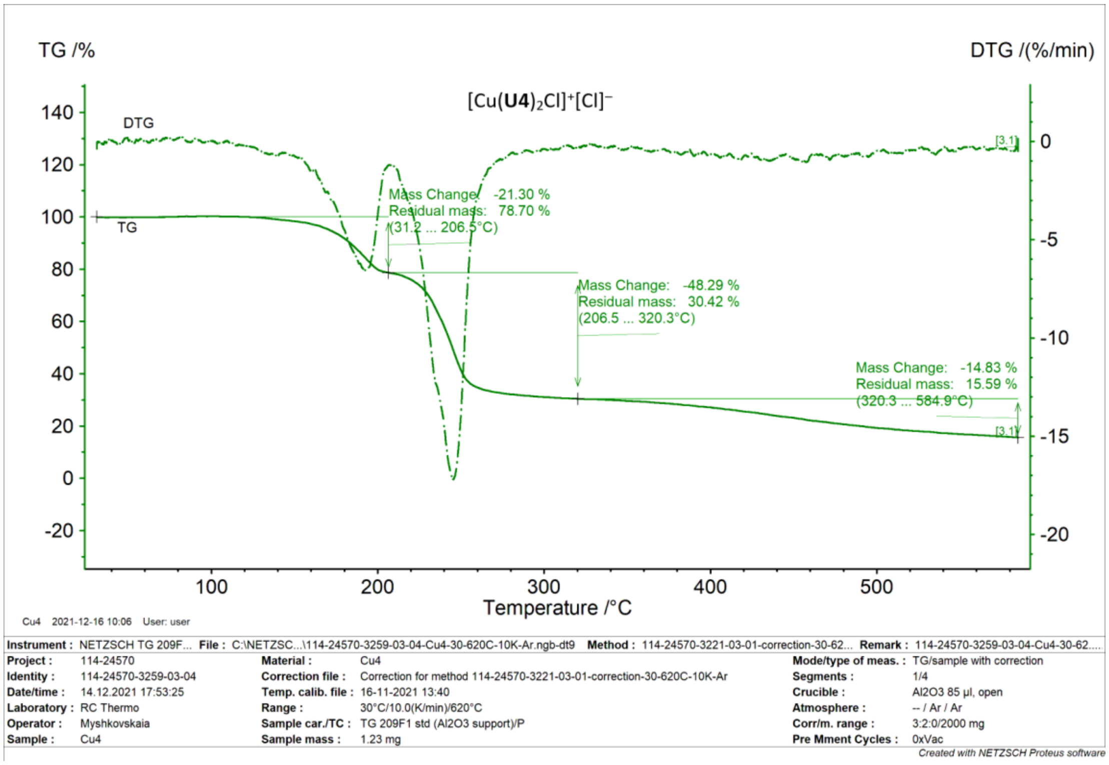

Figure S12. TG/DTG curves of [Cu(**U4**)$_2$Cl]$^+$[Cl]$^-$.

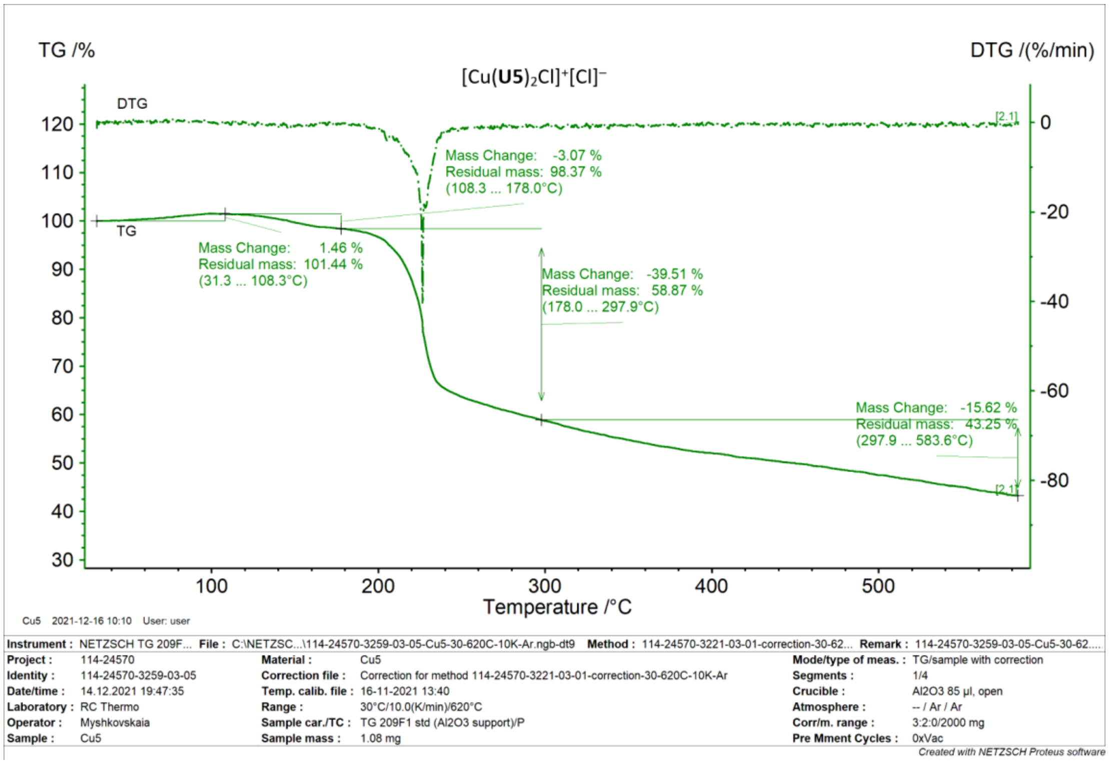

Figure S13. TG/DTG curves of $[Cu(\mathbf{U5})_2Cl]^+[Cl]^-$.

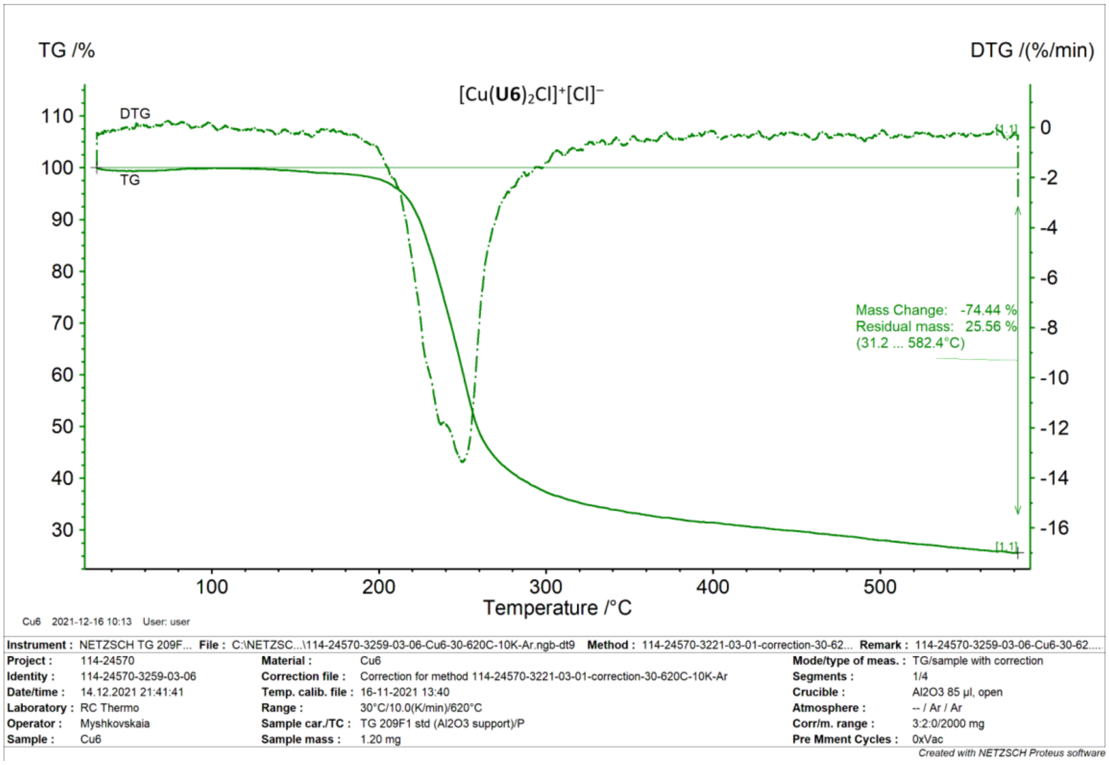

Figure S14. TG/DTG curves of $[Cu(\mathbf{U6})_2Cl]^+[Cl]^-$.

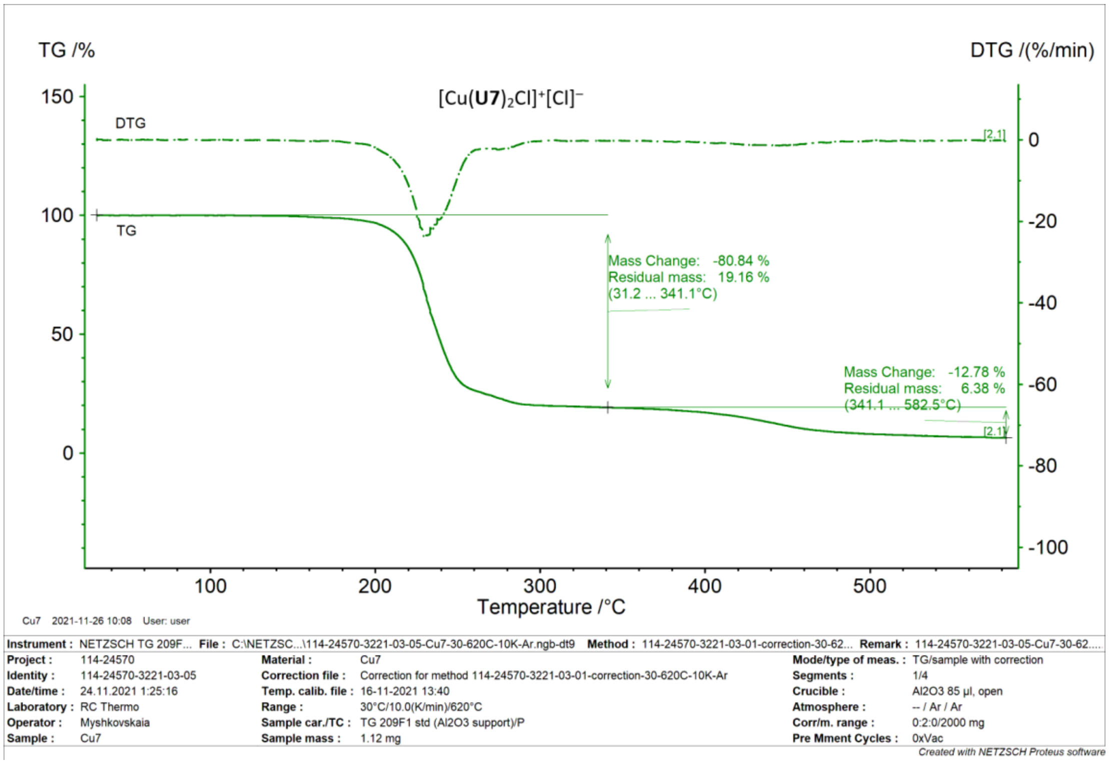

Figure S15. TG/DTG curves of $[Cu(\mathbf{U7})_2Cl]^+[Cl]^-$.

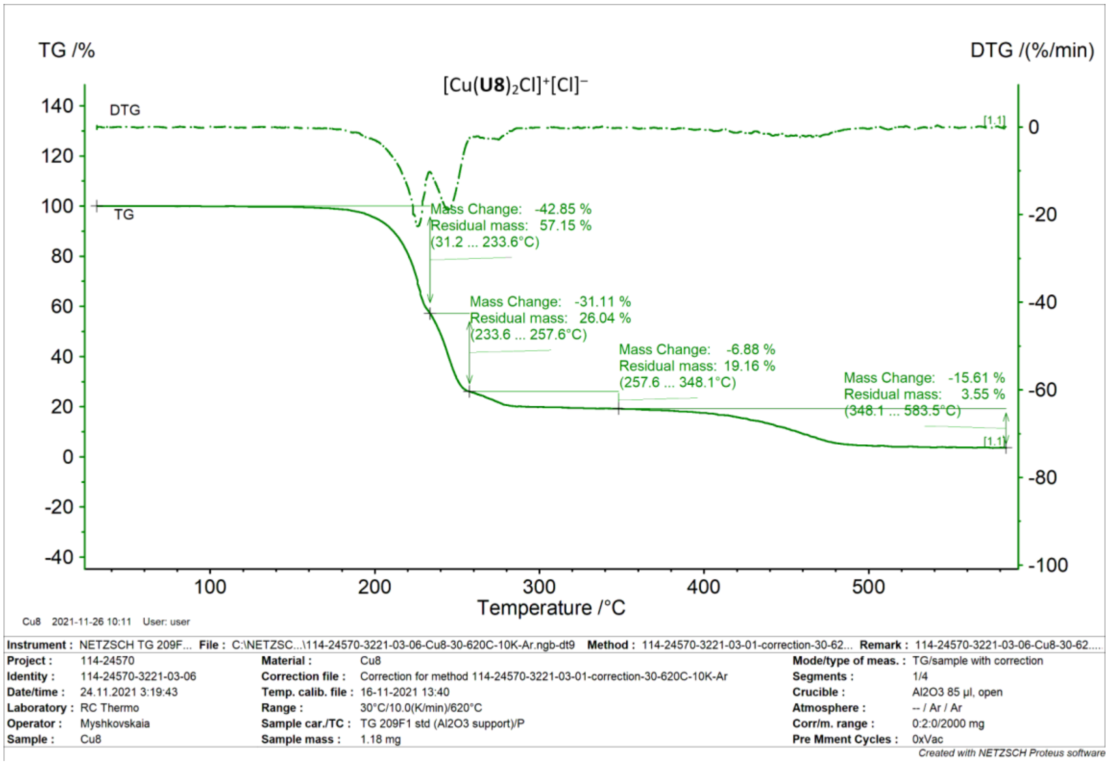

Figure S16. TG/DTG curves of $[Cu(\mathbf{U8})_2Cl]^+[Cl]^-$.

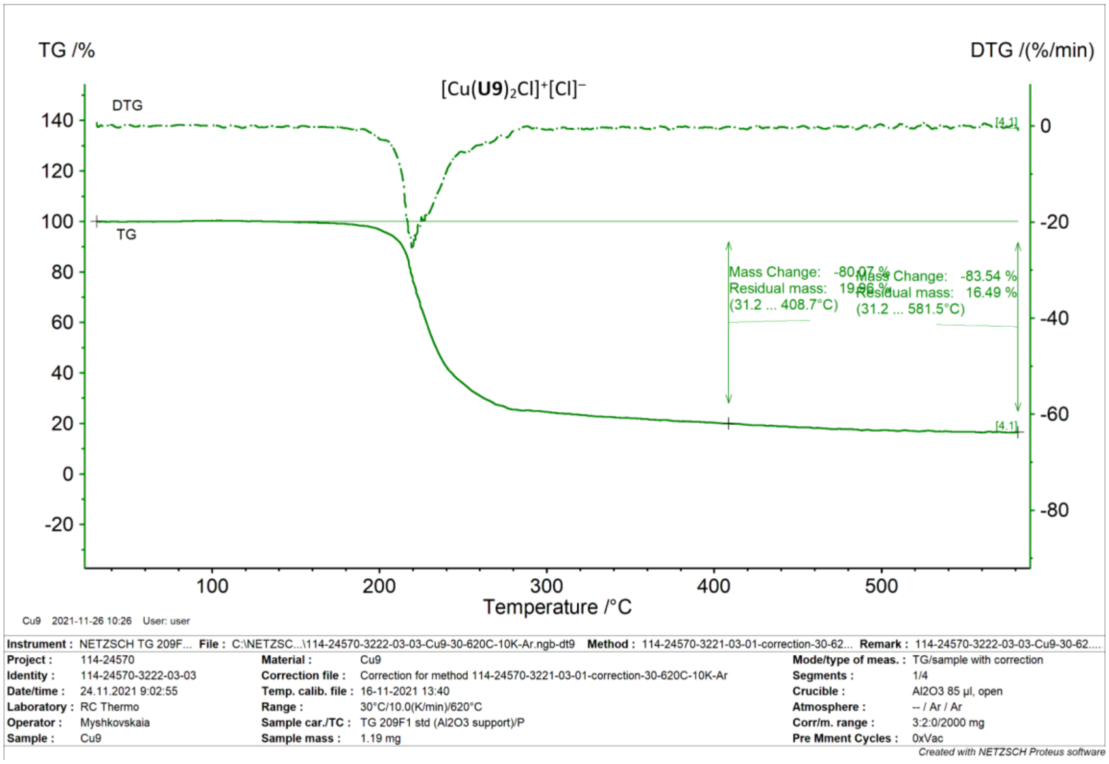

Figure S17. TG/DTG curves of $[Cu(\mathbf{U9})_2Cl]^+[Cl]^-$.

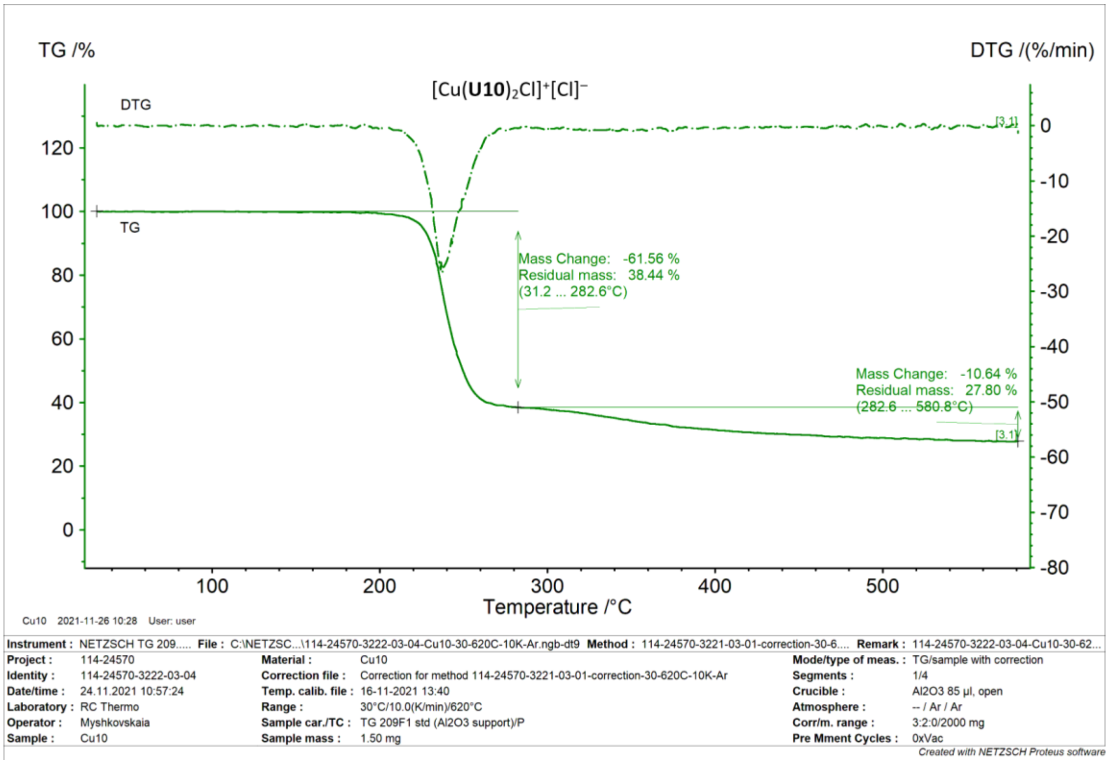

Figure S18. TG/DTG curves of $[Cu(\mathbf{U10})_2Cl]^+[Cl]^-$.

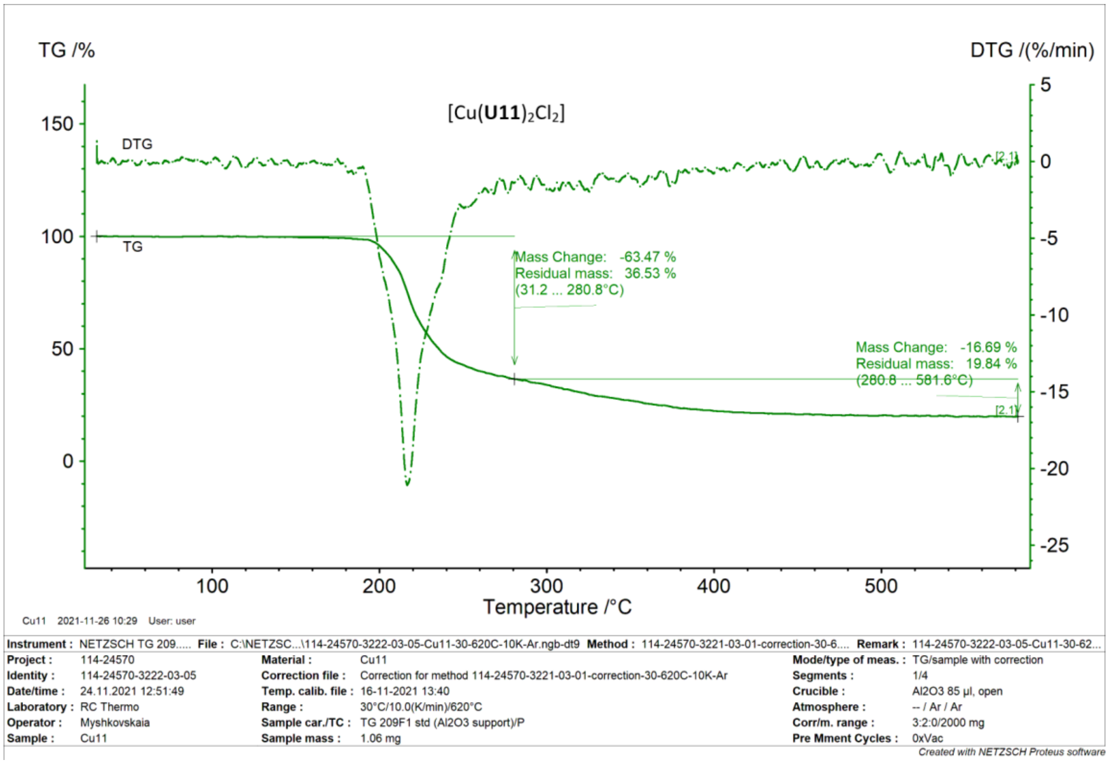

Figure S19. TG/DTG curves of [Cu(**U11**)$_2$Cl$_2$],

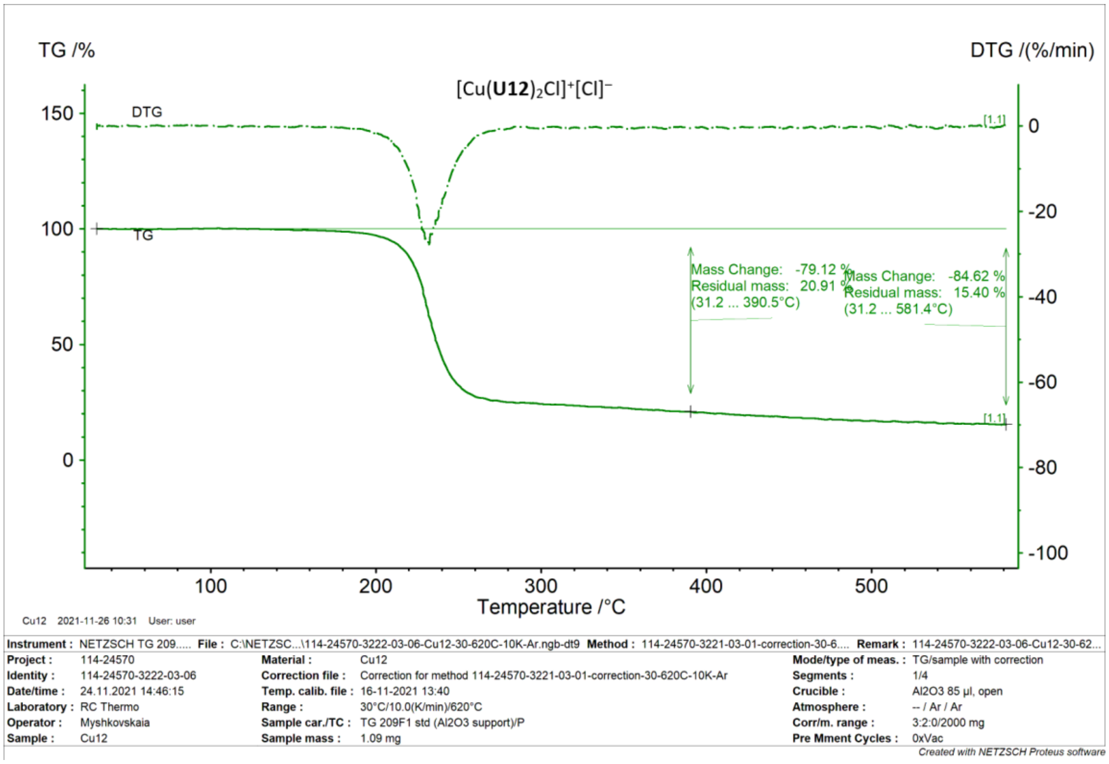

Figure S20. TG/DTG curves of [Cu(**12**)$_2$Cl]$^+$[Cl]$^-$.

4. References

(1) Rassadin, V. A.; Zimin, D. P.; Raskil'dina, G. Z.; Ivanov, A. Y.; Boyarskiy, V. P.; Zlotskii, S. S.; Kukushkin, V. Y. Solvent- and Halide-Free Synthesis of Pyridine-2-Yl Substituted Ureas through Facile C–H Functionalization of Pyridine N-Oxides. *Green Chem.* **2016**, *18* (24), 6630–6636.

(2) Geyl, K.; Baykov, S.; Tarasenko, M.; Zelenkov, L. E.; Matveevskaya, V.; Boyarskiy, V. P. Convenient Entry to N-Pyridinylureas with Pharmaceutically Privileged Oxadiazole Substituents via the Acid-Catalyzed C H Activation of N-Oxides. *Tetrahedron Lett.* **2019**, *60* (40), 151108.

(3) Baykov, S.; Mikherdov, A.; Novikov, A.; Geyl, K.; Tarasenko, M.; Gureev, M.; Boyarskiy, V. π–π Noncovalent Interaction Involving 1,2,4- and 1,3,4-Oxadiazole Systems: The Combined Experimental, Theoretical, and Database Study. *Molecules* **2021**, *26* (18), 5672.

(4) Kasatkina, S. O.; Geyl, K. K.; Baykov, S. V.; Boyarskaya, I. A.; Boyarskiy, V. P. Catalyst-Free Synthesis of Substituted Pyridin-2-Yl, Quinolin-2-Yl, and Isoquinolin-1-Yl Carbamates from the Corresponding Hetaryl Ureas and Alcohols. *Org. Biomol. Chem.* **2021**, *19* (27), 6059–6065.
